# Psychometric precision in phenotype definition is a useful step in molecular genetic investigation of psychiatric disorders

**DOI:** 10.1038/tp.2015.86

**Published:** 2015-06-30

**Authors:** M K Xu, D Gaysina, J H Barnett, L Scoriels, L N van de Lagemaat, A Wong, M Richards, T J Croudace, P B Jones, the LHA genetics group

**Affiliations:** 1 Cardiovascular Epidemiology Unit, Department of Public Health and Primary Care, University of Cambridge, Strangeways Research Laboratory, Cambridge, UK; 2 Rudd Centre for Adoption Research and Practice, School of Psychology, University of Sussex, Brighton, UK; 3 Department of Psychiatry, University of Cambridge, Cambridge, UK; 4 Cambridge Cognition, Cambridge, UK; 5 Instituto de Psiquiatria, Universidade Federal do Rio de Janeiro, Rio de Janeiro, Brazil; 6 Division of Clinical Neuroscience, Edinburgh University, Edinburgh, UK; 7 MRC Unit for Lifelong Health and Ageing at University College London, London, UK; 8 School of Nursing and Midwifery, University of Dundee, Dundee, UK

## Abstract

Affective disorders are highly heritable, but few genetic risk variants have been consistently replicated in molecular genetic association studies. The common method of defining psychiatric phenotypes in molecular genetic research is either a summation of symptom scores or binary threshold score representing the risk of diagnosis. Psychometric latent variable methods can improve the precision of psychiatric phenotypes, especially when the data structure is not straightforward. Using data from the British 1946 birth cohort, we compared summary scores with psychometric modeling based on the General Health Questionnaire (GHQ-28) scale for affective symptoms in an association analysis of 27 candidate genes (249 single-nucleotide polymorphisms (SNPs)). The psychometric method utilized a bi-factor model that partitioned the phenotype variances into five orthogonal latent variable factors, in accordance with the multidimensional data structure of the GHQ-28 involving somatic, social, anxiety and depression domains. Results showed that, compared with the summation approach, the affective symptoms defined by the bi-factor psychometric model had a higher number of associated SNPs of larger effect sizes. These results suggest that psychometrically defined mental health phenotypes can reflect the dimensions of complex phenotypes better than summation scores, and therefore offer a useful approach in genetic association investigations.

## Introduction

Affective disorders are highly heritable,^1, 2, 3^ yet few genetic risk variants have been consistently replicated in molecular genetic association studies.^4, 5^ Recent genome-wide association studies (GWAS) of major depression,^6, 7^ depressive symptoms^8^ and bipolar disorder^9^ have gained substantially stronger statistical power because of increased sample sizes; nonetheless, the majority of genetic variants conferring risk remain unidentified.

Classification of affective disorder is usually based on self-reported symptoms intended to capture psychopathological syndromes through questionnaire responses, clinical ratings and evaluations or by field-trained interviewers. Psychiatric phenotypes defined this way may be especially susceptible to variance introduced by testing methods or other potentially bias-inducing elements that are not primarily related to the disorder itself. Moreover, typical effect sizes of common genetic variants are so small that many existing molecular genetic studies lack sufficient statistical power to detect such small effects. In order to better understand the genetic signals of complex psychiatric phenotypes, there is an argument for adopting approaches that can improve the definition of these phenotyps.^10^ Indeed, as Green *et al.*^11^ noted, ‘even the most precise molecular genetic data cannot be useful if the phenotypes are not well defined'. Some work has previously been done to develop statistical tools for the detection of genetic associations in both candidate gene and GWAS.^10, 12, 13, 14, 15^ For example, van der Sluis *et al.*^14^ have developed the TATES method that combines *P*-values based on correlations between components of multivariate phenotype. The TATES method has demonstrated higher statistical power compared with methods based on composite scores or multivariate analysis of variance.

Many scaled instruments have *a priori* hierarchical and multidimensional structure, with specific clusters of psychiatric signs or symptoms combined into a summation score (for example, the General Health Questionnaire 28-item version (GHQ-28) or the Center for Epidemiological Studies Depression Scale).^16, 17, 18^ The GHQ-28 is a widely used self-report questionnaire to measure psychosocial dysfunction and psychological distress, with the following four subscales: Somatic Symptoms, Anxiety/Insomnia, Social Dysfunction, and Severe Depression.^19, 20^ Traditionally, a single measure of global affective symptoms is derived from item responses and a threshold/cutpoint used to define ‘caseness'. This approach based on summation of items adopts a unidimensional data structure assumption, instead of fully taking into account the dimensions represented by the four subscales. When greater complexity is present, a unidimensional assumption leads to an inaccurate measure of affective symptoms and can alter the strength of association with covariates such as genetic polymorphisms.^21^ Psychometric methods that use latent variable methods to model multidimensional data in intelligence and personality have been developed and are widely used elsewhere.^22, 23, 24^ More recently, these methods have been applied to the understanding of psychiatric phenotypes.^25, 26, 27, 28^ However, they are yet to be widely applied to phenotypes for molecular genetic associations.^29, 30^ Ignoring the complex dimension structure intrinsic to the psychiatric instrument can result in less clear phenotype definition, and may dilute the strength of true genetic association. As a result, it becomes increasingly more difficult to observe consistent association signals for common genetic variants that have small effect sizes.

In the present study using data from a population-representative birth cohort sample, we tested associations between candidate genes previously implicated in affective disorders and alternative outcomes based on two statistical approaches to define affective symptoms measured with the GHQ-28—the traditional sum score approach and the psychometric approach incorporating dimensional aspect of a complex latent phenotype. Rather than seek to identify novel genetic associations, we aim to compare the strength of associations for known candidate genes and phenotypes using standard summation score approach and potentially more accurate psychometric method. The psychometrical approach utilized a latent variable bi-factor model specification.^21, 28^ In the bi-factor model, both the global factor representing total affective symptoms, and the specific factors corresponding to the symptoms of GHQ-28 subdimensions were psychometrically defined using latent variable technique. The bi-factor model is advantageous because it facilitates more flexible phenotype definition in the presence of an *a priori* multidimensional data structure. Hence, we hypothesize that, compared with the sum score approach, the global psychometric latent factor of affective symptoms in the bi-factor model would show stronger genetic associations in terms of effect sizes. Similarly, for specific dimensions of affective symptoms, we hypothesize that the dimension-specific phenotypes defined with the bi-factor models would show stronger genetic associations' effect sizes as compared with the sum score measures for each of these dimensions.

## Materials and methods

### Subjects

The study sample was based on the Medical Research Council National Survey of Health and Development, also known as the British 1946 birth cohort, which originally consisted of 5362 survey members (2547 females and 2815 males) born in 1 week in March 1946 in England, Scotland and Wales.^31^ Both the GHQ-28 data and the blood sample were collected in 1999, when the cohort members were 53 years old. The cohort was shown to be representative of a UK population of the same age.^32^ The data collection received Multi-Centre Research Ethics Committee approval, and participants gave informed consent.

### Phenotype measures

Affective symptoms were assessed with GHQ-28 when the survey members were aged 53 years. Participants (*n*=3035) answered 28 questions, coded on a four-point Likert scale rating from ‘not at all' to ‘much more than usual' on whether they had recently experienced symptoms from four affective disorder domains, i.e. Anxiety/Insomnia (for example, ‘losing sleep over worry'), Somatic Symptoms (for example, ‘feeling ill'), Social Dysfunction (for example, ‘being able to enjoy normal day-to-day activities') and Depression (for example, ‘feeling that life is entirely hopeless'). On the basis of the GHQ-28 responses, phenotype measures of affective symptoms were developed using the following: (1) a traditional sum score approach and (2) a psychometric latent variable approach.

#### Sum score approach

For a global measure of affective symptoms, a Likert sum score based on all 28 GHQ-28 items was calculated. Sum scores were also calculated for the four subscales of affective symptoms, using the items corresponding to each dimension.

#### Psychometric latent variable approach

**Figure 1:**
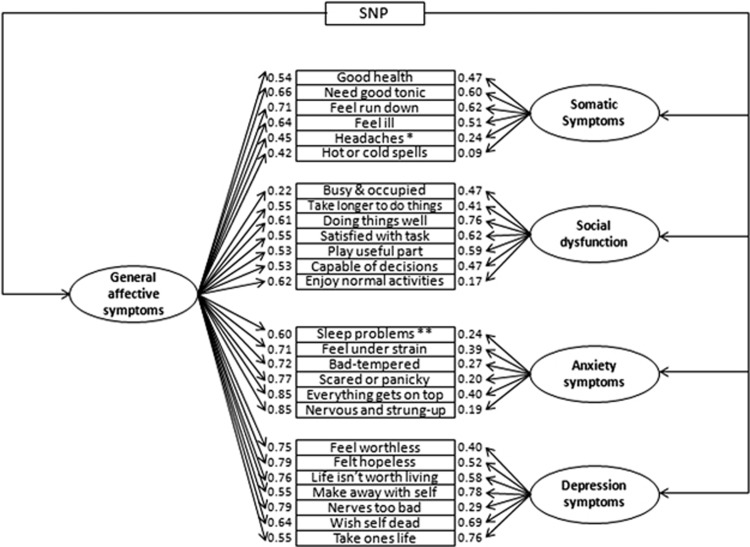
Psychometric model of General Health Questionnaire 28-item version (GHQ-28) items with a single-nucleotide polymorphism (SNP) predictor. Oval shapes are latent variables representing global and specific affective disorder domains. Rectangular shapes are observed variables including both the SNP predictor and GHQ-28 items that are the basis of the latent variables. Arrows leading from a SNP variable to latent variables represent the regression path from the SNP predictor to global as well as specific phenotype dimensions. The arrows between the latent variable to the observed variables indicate the strength of the relationship between the two, represented by standardized factor loadings. The standardized factor loadings are based on a phenotype-only model (excluding the SNP predictor variable from the model). The model fit indices were as follows: 1643.081 (273 degree of freedom) for *X*^2^, 0.061 for Root Mean Square Error of Approximation (RMSEA), 0.968 for Comparative Fit Index (CFI) and 0.961 for Tucker–Lewis Index (TLI). *The indicator ‘headaches' was based on the sum of two highly correlated items. This is to avoid convergence problems caused by the high colinearity between the two items. **Similarly, ‘sleep problems' were also based on two substantially correlated items. Both items were still treated as ordinal measures in model estimation.

The psychometric phenotype of affective symptoms was specified in the form of a bi-factor model (Figure 1). The phenotype outcome variables were posited as latent variables. The bi-factor specification allows the questionnaire items to measure directly both the global factor and four specific factors representing each subdimension of affective symptoms. The factors in the bi-factor model are orthogonal, with covariances among factors specified to be zero. That is, under the bi-factor specification, the global factor is based on information corresponding to the global affective symptom phenotype and is no longer ‘contaminated' by variances that are specific to the subdimensions. Similarly, subdimensions that otherwise embedded in a single global measure according to the sum score approach are now modeled separately as distinct, nonoverlapping dimension-specific latent factors.

### Genotyping

DNA samples (*n*=2756) were extracted and purified from whole blood using the PuregeneDNA Isolation Kit (Flowgen, Leicestershire, UK) according to the manufacturer's protocol.^33^

In total, 249 single-nucleotide polymorphisms (SNPs) covering 27 candidate genes were included in the present study. The candidate genes were selected on the basis of existing evidence for their associations with affective disorders and/or neurocognitive and psychological functions known to be impaired in affective disorders (Supplementary Table S1). Some SNPs of these candidate genes were available from a separate project using the Metabochip.^34, 35^ Additional SNPs of these candidate genes were selected using the Tagger implementation in the Haploview program on the basis of the Hapmap CEU population data to get a better coverage of the gene regions.^36^ SNPs were typed using the KASPar system by LGC, Hoddesdon, UK (http://www.lgcgroup.com/) or Sequenom by Medical Research Council Epidemiology Unit, Cambridge, UK (http://www.sequenom.com/).^34^

The integrity of genotyping was checked with the call rates, concordance of duplicates and minor allele frequency. The call rates for the genotyped SNPs were >90%, with >95% concordance between duplicate samples. Only SNPs with minor allele frequency>0.05 were included in the analysis.

### Statistical analysis

As the candidate genes were chosen on an *a priori* basis, and as our sample was not large enough to allow a high level of power for detection of small effects, we compared effect sizes obtained using the two different phenotype definition approaches by measuring the percentage of phenotype variances explained by a SNP (*R*-squared).

We adopted an additive genetic model for the association analyses. For each genotype, 0, 1 or 2 was assigned for an individual with a homozygote with the major allele, heterozygote and homozygote for the minor allele, respectively.

The final analysis sample consisted of 1337 individuals with complete data. All analyses were performed in the statistical software package Mplus 7.11.^37^ (See Appendix for further details.) For the psychometric analysis based on categorical variables, we used the WLSMV estimator with theta parameterization. Scores for each questionnaire item were modeled as ordered polytomous outcomes, through a probit regression link, to the corresponding latent phenotype variables. This is a graded response, two-parameter normal ogive model in item response theory terms.^38^

### Goodness of fit

In order to evaluate the goodness of fit of the psychometric phenotype model, we presented the *X*^2^-statistic and other commonly used model fit indices. As the *X*^2^-measure is highly sensitive to the sample size, this measure needs to be interpreted with caution.^39, 40^ Other sample size-independent fit indices include the Root Mean Square Error of Approximation (RMSEA), the Tucker–Lewis Index (TLI) and the Comparative Fit Index (CFI).^41, 42, 43^ The TLI and CFI vary along a 0-to-1 continuum, and values greater than 0.90 and 0.95 typically reflect an acceptable and excellent fit to the data. RMSEA values of less than 0.05 and 0.08 reflect a close fit and a minimally acceptable fit to the data, respectively.

We conducted a *post hoc* power analysis for SNPs with the largest effect sizes using statistical simulations. Sample size was fixed at 1337, the same as in the analytic sample. Population parameters were also fixed to be equal to the sample data of the corresponding SNP and phenotype. For each power analysis, 1000 replication data sets were generated and analyzed, with results averaged across the 1000 analysis.

## Results

### Phenotype construction

GHQ item responses were coded using the Likert scale (0-1-2-3). The global mental health sum scores were calculated, ranging from 1 to 65, with a mean score of 17.37. For dimension-specific phenotypes, the average score of somatic symptoms was 4.21, ranging from 0 to 20. For social dysfunction, the average score was 7.14, ranging from 0 to 20. Anxiety scores had a mean of 4.85, with a range of 0–21. The depression sum score ranged from 0 to 19, with an average score of 1.18.

A bi-factor model (Figure 1) was fitted to represent the latent psychometric structure of the phenotype for both global affective symptoms, and the following item sets: somatic symptoms, social dysfunction, anxiety symptoms and depression symptoms. Model fit indices indicated excellent goodness of fit to data (RMSEA: 0.061; CFI: 0.968; TLI: 0.961). All factor loadings were statistically significant at *P*<0.05.

### Genetic association analysis

Association analysis was performed for all SNPs for both the sum score version of the phenotype and the psychometric phenotype (Figure 1). Results for individual SNP analyses are presented in Supplementary Table S2 and Supplementary Table S3.

**Figure 2:**
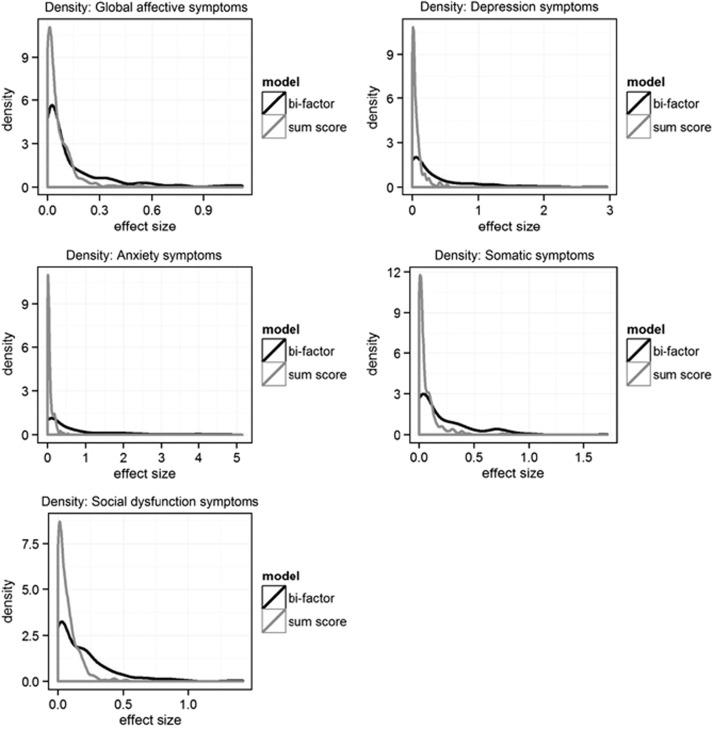
Density plots of single-nucleotide polymorphism (SNP) effect sizes. The *x* axis represents effect size in terms of the percentage of phenotypic variance explained by a single SNP. The *y* axis represents density of effect size.

**Figure 3:**
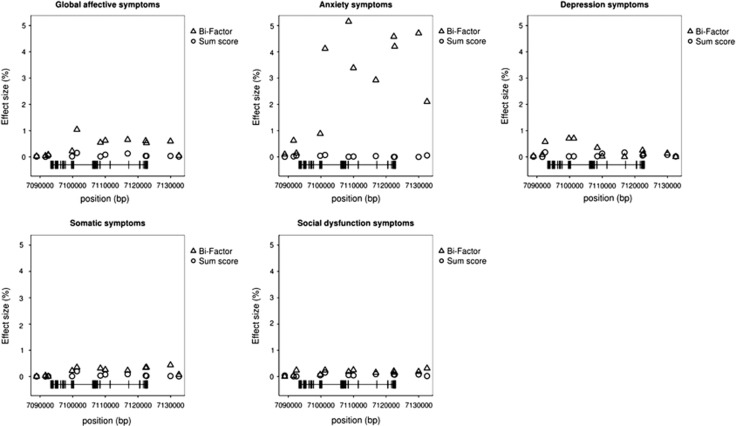
Association results for single-nucleotide polymorphisms (SNPs) in the *DLG4* gene (12 SNPs) for global and specific factor phenotypes. The *y* axis represents effect size in terms of the percentage of variance explained in the phenotype. The *x* axis indicates the chromosome positions (bp). The bars at the bottom of the *x* axis represent exon positions.

Distributions of the effect size frequencies were estimated as densities (Figure 2) and plotted separately for the sum score approach and the bi-factor approach. The area under each curve is 1 and represents the probability of observing an effect size range. As shown in Figure 2, across all phenotype domains, for effect sizes of smaller magnitude, the density of the smallest set of effect sizes was higher for the sum score approach than for the bi-factor approach. In particular, no SNP predictor explained more than 1% of the phenotype variance on the basis of the sum score approach. On the other hand, the density of larger effect sizes was higher for the bi-factor approach. Thus, the bi-factor approach in general yielded a larger number of, and in general, larger effect sizes. To illustrate this finding for a particular candidate gene, the effects of the 12 SNPs of the *DLG4* gene on global affective symptoms and GHQ-28 subscales are shown in Figure 3. Compared with the sum score phenotype, affective symptoms defined by the bi-factor approach had in general larger effect sizes. This was especially the case for the anxiety-symptom subscale.

### *Post hoc* power analysis

*Post hoc* power analysis was performed for five SNPs with the largest effect sizes for global affective disorder, anxiety, depression, social dysfunction and somatic-symptom factors (Table 1). For anxiety-symptom factor, SNP rs1875673 of the *DLG4* gene had effect sizes of 0.006% and 5.153% for the phenotype defined with the sum score approach and bi-factor approach, respectively. The statistical power to detect these effect sizes was only 4.7% for the sum score approach, but was as high as 85.5% for the bi-factor approach. Similar trends favoring the psychometric approach were observed for the other subscales. In particular, in regard to somatic and anxiety symptoms, power based on the sum score approach was below 5%, compared with more than 80% under the bi-factor approach.

### Additional analysis using a model based on four first-order factors

To confirm the analysis with the *DLG4* gene, we tested the SNP association in addition with an alternative psychometric model that specifies four GHQ dimensions as correlated first-order factors (see Supplementary Figure S1). This model implies that the GHQ dimensions are each self-contained constructs. Each dimension is designed to measure an aspect of general mental health; therefore, they are theoretically inter-related and share variances that are because of the common domain of GHQ. In terms of measured breath of phenotype content, the factors in the first-order factor model are comparable to the Likert sum scores of each GHQ dimensions, except that the first-order factor model is able to allow for measurement errors. Results based on the first-order factor model approach (Supplementary Figure S2) were very similar to that of the sum score approach, and the effect sizes of the *DLG4* SNPs remained largest for the bi-factor phenotypic model.

## Discussion

We systematically evaluated the effects of SNPs of candidate genes on affective symptom outcomes defined through traditional sum score and psychometric approaches. In comparison with the sum score approach, the psychometric approach allowed for a more theoretically grounded and statistically refined phenotype definition, and therefore had higher statistical power through larger effect sizes.

The larger effect sizes observed in the bi-factor phenotype analysis were consistent with the application of a more appropriate method for phenotype definition that takes into account the complex structure of the GHQ-28 subscales. The GHQ-28 consists of four affective disorder subscales, each focusing on a specific theme of homogenous symptoms. Although the four subscales are designed to measure a global factor of affective symptoms, each dimension has unique symptom variance that is substantial enough to be modeled as separate factors through a bi-factor specification. As the bi-factor model distinguishes the global factor from the specific subscale factors, it allows the possibility of detecting genetic associations with higher level of phenotype precision that would otherwise be difficult to detect (that is, using the sum score approach).

The sum score of depression items had the highest skewness (3.78 compared with between 1 and 1.5 for skewness from other domains) with a rather low mean of 1.18 out of a possible highest score of 21. Reduced power is present in data that are highly skewed; nevertheless, latent variable approach can improve the power of such data.^10^

In the present study, some genetic variants were shown to be associated with specific subscales of affective symptoms measured by GHQ-28. This pattern of results was most clearly shown under the bi-factor model. For example, the effect of the *DLG4* gene on anxiety symptoms emerged only under the bi-factor model method. Compelling evidence suggests that affective disorders are associated with dysfunction of brain glutamatergic transmission.^44^ The *DLG4* gene, which encodes post-synaptic density protein 95 (PSD95), has a critical role in regulating *N*-methyl-d-aspartate receptor receptor activity and its signal transduction; PSD95 is a member of the synapse-associated protein family of scaffolding molecules that control the organization, composition and function of synapses.^45, 46^ In particular, *DLG4* knockout mice showed increased repetitive behaviors, abnormal communication and social behaviors, impaired motor coordination and increased stress reactivity and anxiety-related responses.^47^ Taken together, these findings indicate that the aberrant expression and function of PSD95 may contribute to the compromised *N*-methyl-d-aspartate receptor-mediated signaling in affective symptoms, and in particular anxiety and stress responses.

The heritability of domains and global measures of the GHQ-28 was previously studied in a representative twin sample from the United Kingdom.^48^ Although the heritability of global affective symptoms was 44%, the percentage varied from 20% for social dysfunction to 44% for depression, indicating heterogeneity of the genetic underpinnings of the subscales. A more recent twin study examined the role of genetic factors in neuroticism, anxiety/depression and somatic distress.^49^ The study estimated that anxiety/depression shared 11% genetic variance with somatic stress. These findings demonstrate that even closely related subscales of affective symptoms can have unshared, unique genetic variances. Failure to incorporate the dimensionality aspect of complex traits could lead to reduced power to detect genetic associations.^21^ This has also been investigated in simulation studies of twin designs.^13^

In terms of association analysis on the basis of the first-order model, whereas the first-order model reflected the dimensionality of the GHQ structure on the subscale level, it failed to take into account the global factor that represents global mental health. The bi-factor model, on the other hand, was able to overcome the dimensionality limitations of the first-order model and allowed for both a global factor as well as specific factors, which are essentially variances after taking into account of the global factor. The factors in the bi-factor model are all orthogonal to each other (covariances among factors are specified to be zero), reflecting an implied assumption that the global factor explains the correlations among the subdimensions. That is, the general mental health phenotype is only based on information that is no longer ‘contaminated' by variances from the specific dimensions; similarly, traits that originally overlap with each subdimension are modeled separately as distinct, nonoverlapping factors. The unique association between the *DLG4* gene and specific factor on anxiety indicates that the *DLG4* potentially contributes to mental health symptoms mainly through anxiety symptoms that are not shared by other dimensions of the GHQ. If the ultimate purpose was to reduce global mental health symptoms (in contrast to specific factors for each symptom dimension), then targeting the *DLG4* gene would not be an efficient route. However, for better understanding of severe anxiety disorder, it could be fruitful to explore *DLG4* biochemical pathways.

The advantage of the bi-factor model specification is that it fully takes into account a global domain as well as the specific domains in the presence of data complexity/multidimensionality. For multidimensional phenotypes, a definition that emphasizes unidimensionality (such as using a single sum score to represent a global measure) produces a less accurate phenotype definition, thus underestimating the strength of association between a SNP and a phenotype of interest. This can consequently lead to reduced statistical power in detecting associations because the genetic link is masked by variances due to specific dimensions embedded in the phenotype measure. The bi-factor specification resolves this issue by separating the variances into phenotypes that are specific to both the global construct and the specific dimensions. Moreover, as the associations between a SNP and a phenotype of interest are simultaneously estimated in a latent variable framework, measurement errors are taken into account so as to enable more accurate genetic association estimation.

It is worth noting that, even under the bi-factor psychometric method, genetic association analyses using the global affective symptom factor had weaker effect sizes than the subdimensions of affective symptoms. The disparity in the densities of SNPs with relatively low effect sizes between the sum score and bi-factor approach is less pronounced for the global factor compared with the differences between the two approaches in specific factors. This indicates genetic heterogeneity of the global factor from specific factors. It seems that the global, common mental health factor is less explained by the candidate gene SNPs included in the present studies. Instead of focusing on a global phenotype that highlights shared common variances among all dimension-specific symptoms, it might be more informative to look at dimension-specific phenotypes that are conceptually more homogeneous. The genetic architecture of dimension-specific phenotypes could be simpler than that of the broader global construct.

Although in the present investigation the bi-factor model fitted the data very well, the factor structure is specified *a priori*, and correlations between global and specific factors were specified to be orthogonal. This was because the GHQ-28 is an instrument with predefined symptom dimensions. In situations when the data structure is not known or not well established, it is possible to relax this specification and use a more exploratory framework, allowing dimensions to be correlated through bi-factor rotation methods.^50, 51, 52^

Psychometric approaches can be applicable in a variety of contexts in psychiatric genetics and beyond, where the data structure of phenotypes of interests is complex. For example, data collected from studies that utilize different forms of assessment methods might be prone to method effects.^53, 54, 55^ A phenotype might consist of symptom items assessed by nurses, family members and self-completed questionnaires. Although the effect of an external assessor or self-administration should be irrelevant to the association between the target phenotype and genetic predictors, variances introduced by these unique external sources potentially contaminate the phenotype measure and prevent accurate genetic signals being observed.

Another issue is that, although this type of analysis is fast when only several dozens of SNPs are included, it would require substantial computational power to test for genome-wide associations with hundreds of thousands of SNPs.

It is likely that the relationship between genetic and phenotypic variables is much more complex. For example, there could be a direct effect from a SNP variable to the individual items even after accounting for the association between the SNP and the latent phenotypic variable. In particular, van de Sluis *et al.*^14^ investigated how to approach gene finding research if the exact location of a SNP effect in multivariate data was unknown. In addition, when subgroups of individuals (for example, males and females) are present in the data, it is possible that the measurement model of the phenotypes might differ across these groups, as will the association between a genetic variant and a phenotype. Ignoring hidden genetic–phenotype structures can lead to biased findings. Some previous studies have investigated the impact that phenotype measurement bias can have on the power to detect genetic variants.^14, 15, 56^ These topics are beyond the scope of the analytic framework in the present investigation, but need to be explored in future studies.

In conclusion, the psychometric approach for phenotype definition represents a useful step for genetic investigations of psychiatric disorders. This approach affords a more flexible phenotype definition, and thus provides greater statistical strength to detect genetic associations.

## Figures and Tables

**Table 1:** Power analysis of sum score and bi-factor approaches for SNPs with largest effect size in bi-factor association results

*Method*	*Global affective symptoms*		*Anxiety symptoms*		*Depression symptoms*		*Social dysfunction*		*Somatic symptoms*	
	*Sum score*	*Psychometric*	*Sum score*	*Psychometric*	*Sum score*	*Psychometric*	*Sum score*	*Psychometric*	*Sum score*	*Psychometric*
SNP	rs2793085		rs1875673		rs6603803		rs11233640		rs2070951	
Gene	*DSC1*		*DLG4*		*GNB1*		*DLG2*		*NR3C2*	
Effect size (%)	0.53	1.12	0.01	5.15	0.40	2.96	0.30	1.42	0.03	1.72
Power (%)	76.10	79.50	4.70	85.50	63.90	78.50	54.00	80.20	10.10	83.70

Abbreviation: SNP, single-nucleotide polymorphism.

Effect size is based on percentage of explained phenotype variances by a single SNP predictor.

Power statistic is based on 1000 simulated replications; sample size was fixed at 1337 as in the current sample.

## Appendix

### Mplus input syntax, GHQ bi-factor phenotypes predicted by a single SNP

TITLE: Genetic association analysis of SNP rs1875673 and GHQ factors modeled with bi-factor model;

DATA:

FILE IS data.dat;

VARIABLE:

NAMES ARE

ghq0199 ghq0299 ghq0399 ghq0499 ghq0599 ghq0699 ghq0799 ghq0899 ghq0999

ghq1099 ghq1199 ghq1299 ghq1399 ghq1499 ghq1599 ghq1699 ghq1799 ghq1899 ghq1999

ghq2099 ghq2199 ghq2299 ghq2399 ghq2499 ghq2599 ghq2699 ghq2799 ghq2899

rs1875673;

USEVARIABLES ARE

v233 ghq0199 ghq0299 ghq0399 ghq0499 ghq0799

ghq1099 ghq1199 ghq1299 ghq1399 ghq1499 ghq1599 ghq1699 ghq1799

ghq1899 ghq1999 ghq2099 ghq2199 ghq2299 ghq2399 ghq2499 ghq2599 ghq2699 ghq2799

ghq2899 ghq5a6 ghq8a9;

CATEGORICAL ARE

ghq0199 ghq0299 ghq0399 ghq0499 ghq0799

ghq1099 ghq1199 ghq1299 ghq1399 ghq1499 ghq1599 ghq1699 ghq1799

ghq1899 ghq1999 ghq2099 ghq2199 ghq2299 ghq2399 ghq2499 ghq2599 ghq2699 ghq2799

ghq2899 ghq5a6 ghq8a9;

DEFINE:

ghq5a6=SUM (ghq0599 ghq0699);

ghq8a9=SUM (ghq0899 ghq0999);

ANALYSIS:

PARAMETERIZATION=THETA;

MODEL:

MENTAL by

ghq8a9 GHQ1699 GHQ1899 GHQ1999 GHQ2099 GHQ2399

GHQ2199 GHQ2299 GHQ2499 GHQ2599 GHQ2699 GHQ2799 GHQ2899

GHQ1099 GHQ1199 GHQ1299 GHQ1399 GHQ1499 GHQ1599 GHQ1799

GHQ0199 GHQ0299 GHQ0399 GHQ0499 ghq5a6 GHQ0799;

SPANX by

ghq8a9 GHQ1699 GHQ1899 GHQ1999 GHQ2099 GHQ2399;

SPDEP by

GHQ2199 GHQ2299 GHQ2499 GHQ2599 GHQ2699 GHQ2799 GHQ2899;

SPSOC by

GHQ1099 GHQ1199 GHQ1299 GHQ1399 GHQ1499 GHQ1599 GHQ1799;

SPSOM by

GHQ0199 GHQ0299 GHQ0399 GHQ0499 ghq5a6 GHQ0799;

MENTAL WITH SPANX@0 SPDEP@0 SPSOC@0 SPSOM@0;

SPANX WITH SPDEP@0 SPSOC@0 SPSOM@0;

SPDEP WITH SPSOC@0 SPSOM@0;

SPSOC WITH SPSOM@0;

SPANX ON rs1875673;

SPDEP ON rs1875673;

SPSOC ON rs1875673;

SPSOM ON rs1875673;

MENTAL ON rs1875673;
