# Supplementary material for: Psychometric precision in phenotype definition is a useful step in molecular genetic investigation of psychiatric disorders
Source: Transl Psychiatry. 2015 Jun 30;5(6):e593–. doi: 10.1038/tp.2015.86 (PMC4490295; doi:10.1038/tp.2015.86)
Supplement: Supplementary Table 1 [file tp201586x1.doc]

Table S1. Candidate genes for affective disorders

| **Gene, location** | **SNP** | **Position** | **Gene function** | **References** |
| --- | --- | --- | --- | --- |
| ***ACE***  17q23 | rs1800764 | 61550529 | Angiotensin I converting enzyme 1 is a part of the renin–angiotensin system and is involved in the conversion of angiotensin I into the most biologically active product angiotensin II (AT II). AT II is a peptide hormone which acts as a stimulator of proinflammatory cytokines and interferes with HPA-axis activation in response to stress. | [^1-3^](#_ENREF_1) |
|  | rs4291 | 61554194 |  |  |
|  | rs4305 | 61558229 |  |  |
|  | rs4324 | 61563171 |  |  |
|  | rs4353 | 61570422 |  |  |
|  | rs4461142 | 61578048 |  |  |
|  | rs4267385 | 61583756 |  |  |
|  | rs496859 | 89236496 |  |  |
| ***APP***  21q21.3 | rs7277612 | 27257859 | Amyloid precursor protein is an integral membrane protein expressed in many tissues and concentrated in the synapses of neurons. It has been implicated as a regulator of synapse formation, neural plasticity and iron export. APP is the precursor molecule for the beta amyloid, the primary component of amyloid plaques. | [^4^](#_ENREF_4) |
|  | rs17001492 | 27278247 |  |  |
|  | rs2829981 | 27280696 |  |  |
|  | rs4817076 | 27354060 |  |  |
|  | rs2830012 | 27397607 |  |  |
|  | rs3787644 | 27419070 |  |  |
|  | rs2830034 | 27431612 |  |  |
| ***BAIAP2***  17q25 | rs4969361 | 79020533 | Brain-specific angiogenesis inhibitor (BAI1)-binding protein functions as an insulin receptor tyrosine kinase substrate, which may be effective in the central nervous system. It has also been associated with the formation of stress fibers and cytokinesis and may be involved in neuronal growth-cone guidance. |  |
|  | rs8080252 | 79027296 |  |  |
|  | rs12937996 | 79029129 |  |  |
|  | rs4969245 | 79056453 |  |  |
|  | rs12944983 | 79079293 |  |  |
| ***BDNF***  11p14 | rs6265 | 27679916 | Brain derived neurotrophic factor is a secretory protective protein important in neuronal differentiation, survival and plasticity. BDNF plays an important role in the activity-dependent regulation of synaptic structure and function, particularly of the glutamatergic synapses. | [^7-10^](#_ENREF_7) |
|  | rs11030101 | 27680744 |  |  |
|  | rs11030107 | 27694835 |  |  |
|  | rs7103411 | 27700125 |  |  |
|  | rs7127507 | 27714884 |  |  |
| ***CADM1***  11q23.2 | rs1048932 | 115044850 | CADM1 is involved in the immune response of tumor cells. It is essential for the development and survival of mast cells and it may mediate attachment to and promote communication with nerves. The protein also acts as a synaptic cell adhesion molecule and plays a role in the formation of dendritic spines and in synapse assembly; it has also been shown to be involved in neuronal migration, axon growth, path finding, and fasciculation on the axons of differentiating neurons. | [^11-13^](#_ENREF_11) |
|  | rs17118125 | 115114732 |  |  |
|  | rs10502199 | 115120615 |  |  |
| ***CAMK2A***  5q32 | rs2241695 | 149602824 | CAMK2A is important for long-term potentiation and plasticity at glutamatergic synapses. It is part of the post-synaptic density proteins; Ca2+ influx through NMDA receptors activates CAMK2A and its translocation to the NR2B NMDA receptor subunit phosphorylation site | [^14^](#_ENREF_14) |
|  | rs4958469 | 149605116 |  |  |
|  | rs2053053 | 149609393 |  |  |
|  | rs3756578 | 149625087 |  |  |
|  | rs7711562 | 149627423 |  |  |
|  | rs2288799 | 149631413 |  |  |
|  | rs4958445 | 149638360 |  |  |
|  | rs4958902 | 149648780 |  |  |
|  | rs919740 | 149665849 |  |  |
|  | rs11167499 | 149708571 |  |  |
|  | rs2295223 | 149770058 |  |  |
| ***COMT***  22q11.2 | rs7289747 | 19910716 | Catechol-O-methyltransferase methylates a wide range of catechol substrates, including catecholamines. | [^15-17^](#_ENREF_15) |
|  | rs737865 | 19930121 |  |  |
|  | rs6269 | 19949952 |  |  |
|  | rs4633 | 19950235 |  |  |
|  | rs4818 | 19951207 |  |  |
|  | rs4680 | 19951271 |  |  |
|  | rs165599 | 19956781 |  |  |
| ***DAOA***  13q33.2 | rs778294 | 106142235 | D-amino acid oxidase activator activates DAO, which is involved in the conversion of D-serine into pyruvate. D-Serine is an important regulator of the N-methyl D-aspartate (NMDA)-type glutamate receptors in brain and has an important role in neuronal migration and synaptic plasticity. | [^18-20^](#_ENREF_18) |
|  | rs3918342 | 106185749 |  |  |
|  | rs1421292 | 106198235 |  |  |
| ***DISC1***  1q42.1 | rs1059595 | 231658301 | Disrupted-In-Schizophrenia-1 is highly expressed during brain development and plays vital role in the growth of the embryonic and postnatal brain. It regulates neurogenesis via Wnt/β-catenin signalling. | [^21-23^](#_ENREF_21) |
|  | rs2570409 | 231731631 |  |  |
|  | rs3738401 | 231830295 |  |  |
|  | rs1934909 | 231851279 |  |  |
|  | rs12027635 | 231873198 |  |  |
|  | rs2793085 | 231899877 |  |  |
|  | rs1322783 | 231929075 |  |  |
|  | rs6675281 | 231954101 |  |  |
|  | rs9431708 | 232009402 |  |  |
|  | rs7541019 | 232051185 |  |  |
|  | rs6670775 | 232096701 |  |  |
|  | rs821597 | 232102264 |  |  |
|  | rs821616 | 232144598 |  |  |
|  | rs11802446 | 232180614 |  |  |
| ***DLG2***  11q14.1 | rs891773 | 83175779 | DLG2 is involved in the postsynaptic sites to form a multimeric scaffold for the clustering of receptors, ion channels, and associated signaling proteins. It is required for perception of chronic pain through NMDA receptor signaling. It regulates surface expression of NMDA receptors in dorsal horn neurons of the spinal cord. Interacts with the cytoplasmic tail of NMDA receptor subunits as well as inward rectifying potassium channels. Involved in regulation of synaptic stability at cholinergic synapses. |  |
|  | rs11233640 | 83201073 |  |  |
|  | rs11233649 | 83221390 |  |  |
|  | rs3815988 | 83244032 |  |  |
|  | rs11233660 | 83248167 |  |  |
|  | rs6592123 | 83257902 |  |  |
|  | rs6592124 | 83258042 |  |  |
|  | rs485199 | 83334502 |  |  |
|  | rs7945102 | 83440261 |  |  |
|  | rs7126807 | 83525211 |  |  |
|  | rs1037353 | 83525588 |  |  |
|  | rs1400313 | 83534623 |  |  |
|  | rs4480568 | 83537007 |  |  |
|  | rs7926468 | 83541199 |  |  |
|  | rs10792694 | 83542182 |  |  |
|  | rs10898159 | 83555289 |  |  |
|  | rs10898161 | 83560414 |  |  |
|  | rs1483408 | 83673295 |  |  |
|  | rs7125294 | 84111645 |  |  |
|  | rs11824888 | 84318450 |  |  |
|  | rs7129994 | 84380034 |  |  |
|  | rs11234131 | 84401940 |  |  |
|  | rs548098 | 84449873 |  |  |
|  | rs11234161 | 84469196 |  |  |
|  | rs10898305 | 84546912 |  |  |
|  | rs1943711 | 84555967 |  |  |
|  | rs1943733 | 84600597 |  |  |
| ***DLG4***  17p13.1 | rs11650232 | 7088923 | DLG4, together with DLG2 protein, interacts at postsynaptic sites to form a multimeric scaffold for the clustering of receptors, ion channels, and associated signaling proteins. It is required for synaptic plasticity associated with NMDA receptor signaling. |  |
|  | rs314253 | 7091650 |  |  |
|  | rs314252 | 7092506 |  |  |
|  | rs17203281 | 7099811 |  |  |
|  | rs3826408 | 7101292 |  |  |
|  | rs1875673 | 7108506 |  |  |
|  | rs390200 | 7109995 |  |  |
|  | rs446994 | 7116853 |  |  |
|  | rs739669 | 7122377 |  |  |
|  | rs2017365 | 7122624 |  |  |
|  | rs2074222 | 7129974 |  |  |
|  | rs222837 | 7132556 |  |  |
| ***DLGAP2***  8p23 | rs4876080 | 1460796 | DLGAP2 plays a role in synapse organization and signaling in neuronal cells. It may also play a role in the link between ion channel and the subsynaptic cytoskeleton, and it has been found that this protein induces enrichment of post-synaptic density proteins at the plasma membrane. |  |
|  | rs2019513 | 1490055 |  |  |
|  | rs2956913 | 1512528 |  |  |
|  | rs10448097 | 1603043 |  |  |
|  | rs6992443 | 1649938 |  |  |
| ***DLGAP4***  20q11.23 | rs6124857 | 35051207 | DLGAP4 found at the postsynaptic density in neuronal cells. It is a signaling molecule that can interact with potassium channels and receptors, as well as other signaling molecules. This protein interacts with proteins from the postsynaptic density and may be involved in clustering PSD-95 in the postsynaptic density region. | [^30^](#_ENREF_30) |
| ***FKBP5***  6p21.3-p21.2 | rs3800374 | 35537406 | FK506 binding protein is a modulator of glucocorticoid receptor function through association with heat shock protein 90, a molecular chaperone with a central role in steroid hormone signalling. | [^31-33^](#_ENREF_31) |
|  | rs10807151 | 35540868 |  |  |
|  | rs3800373 | 35542476 |  |  |
|  | rs7757037 | 35548236 |  |  |
|  | rs9380524 | 35589070 |  |  |
|  | rs7748266 | 35592744 |  |  |
|  | rs1360780 | 35607571 |  |  |
|  | rs1334894 | 35615130 |  |  |
|  | rs9380525 | 35633038 |  |  |
|  | rs9368881 | 35634288 |  |  |
|  | rs4713916 | 35669983 |  |  |
|  | rs2395635 | 35671165 |  |  |
|  | rs2766534 | 35685714 |  |  |
| ***GNB1***  1p36.33 | rs9660180 | 1723031 | GNB1 is involved as a modulator in various transmembrane signaling systems. It is involved in energy production related to GTP. | [^34^](#_ENREF_34) |
|  | rs6603803 | 1812688 |  |  |
| ***GRIA1***  5q31.1 | rs1864205 | 152884830 | GRIA1 is the AMPA ionotropic glutamate receptor, and via the modulation of cation channels, converts the chemical signal to an electrical impulse. | [^35^](#_ENREF_35) |
|  | rs7727515 | 152892304 |  |  |
|  | rs9324750 | 152916029 |  |  |
|  | rs12522802 | 152942223 |  |  |
|  | rs12515520 | 152955663 |  |  |
|  | rs17519810 | 152963720 |  |  |
|  | rs1994862 | 152988910 |  |  |
|  | rs1422884 | 153009489 |  |  |
|  | rs10515697 | 153010645 |  |  |
|  | rs2963954 | 153024581 |  |  |
|  | rs707176 | 153029960 |  |  |
|  | rs2910258 | 153030802 |  |  |
|  | rs4385264 | 153053735 |  |  |
|  | rs7714428 | 153055075 |  |  |
|  | rs11741511 | 153058142 |  |  |
|  | rs4077374 | 153058885 |  |  |
|  | rs17594729 | 153063802 |  |  |
|  | rs11953799 | 153095004 |  |  |
|  | rs10035143 | 153095304 |  |  |
| ***GRIN2A***  16p13.2 | rs1014531 | 9855794 | GRIN2A encodes for a subunit of the NMDA ionotropic glutamate receptor. When activated, the receptor activates several signaling cascades, including pathways leading to the induction of long-term potentiation and depression. NMDA receptors have a critical role in excitatory synaptic transmission and plasticity in the CNS. They govern a range of physiological conditions including neurological disorders caused by excitotoxic neuronal injury, psychiatric disorders and neuropathic pain syndromes. |  |
|  | rs7190785 | 9873465 |  |  |
|  | rs1544604 | 9887244 |  |  |
|  | rs8050843 | 9930580 |  |  |
|  | rs2215718 | 9938796 |  |  |
|  | rs1548808 | 9966207 |  |  |
|  | rs9931155 | 9984523 |  |  |
|  | rs4782039 | 10006967 |  |  |
|  | rs13331097 | 10052349 |  |  |
|  | rs3859123 | 10070895 |  |  |
|  | rs837694 | 10110040 |  |  |
|  | rs11074568 | 10176042 |  |  |
|  | rs10438517 | 10247304 |  |  |
|  | rs7499321 | 10270314 |  |  |
|  | rs1420666 | 10279848 |  |  |
| ***GRIN2B***  12p12 | rs1806213 | 13723977 | GRIN2B encodes for a subunit of the NMDA ionotropic glutamate receptor. When activated, the receptor activates several signaling cascades, including pathways leading to the induction of long-term potentiation and depression. NMDA receptors have a critical role in excitatory synaptic transmission and plasticity in the CNS. They govern a range of physiological conditions including neurological disorders caused by excitotoxic neuronal injury, psychiatric disorders and neuropathic pain syndromes. |  |
|  | rs1805513 | 13753649 |  |  |
|  | rs12321321 | 13844423 |  |  |
|  | rs2300250 | 13860621 |  |  |
|  | rs4763358 | 13873691 |  |  |
|  | rs1075010 | 13918341 |  |  |
|  | rs7301500 | 13941779 |  |  |
|  | rs17833967 | 13955078 |  |  |
|  | rs10772719 | 14068697 |  |  |
|  | rs12824372 | 14121660 |  |  |
|  | rs17339365 | 14129416 |  |  |
| ***GRM3***  7q21.1-q21.2 | rs802432 | 86284433 | GRM3 belongs to the metabotropic class of glutamate receptors. These receptors inhibit adenylyl cyclase, decreasing the formation of cAMP. They are involved in presynaptic inhibition and do not appear to affect postsynaptic membrane potential by themselves. |  |
|  | rs802434 | 86285544 |  |  |
|  | rs2228595 | 86415987 |  |  |
|  | rs1468412 | 86433451 |  |  |
|  | rs2282966 | 86475667 |  |  |
| ***IQSEC3***  12p13.33 | rs7294904 | 199532 | IQSEC3 acts as a guanine nucleotide exchange factor for ARF1. | [^40^](#_ENREF_40) |
|  | rs2368785 | 260682 |  |  |
| ***LRRC7***  1p31.1 | rs1340769 | 70219936 | LRRC7 is required for normal synaptic spine architecture and function. Necessary for DISC1 and GRM5 localization to postsynaptic density complexes and for both N-methyl D-aspartate receptor-dependent and metabotropic glutamate receptor-dependent long term depression. | [^41^](#_ENREF_41) |
|  | rs6697066 | 70278764 |  |  |
|  | rs10789303 | 70278987 |  |  |
|  | rs1913268 | 70279860 |  |  |
|  | rs12033206 | 70558599 |  |  |
|  | rs12136628 | 70571077 |  |  |
| ***NR3C1***  5q31.3 | rs17209237 | 142657212 | NR3C1 encodes glucocorticoid receptor. It can function both as a transcription factor that binds to glucocorticoid response elements in the promoters of glucocorticoid responsive genes to activate their transcription, and as a regulator of other transcription factors. It is involved in inflammatory responses, cellular proliferation, and differentiation in target tissues. | [^42-45^](#_ENREF_42) |
|  | rs6196 | 142661490 |  |  |
|  | rs852977 | 142687494 |  |  |
|  | rs860457 | 142688323 |  |  |
|  | rs10482689 | 142693032 |  |  |
|  | rs41423247 | 142778575 |  |  |
|  | rs10482605 | 142783521 |  |  |
|  | rs10052957 | 142786701 |  |  |
| ***NR3C2***  4q31 | rs17620330 | 149097709 | NR3C2 encodes the mineralocorticoid receptor, which mediates aldosterone actions on salt and water balance within  restricted target cells. The protein functions as a ligand-dependent transcription factor that binds to  mineralocorticoid response elements in order to trans-activate target genes. | [^46^](#_ENREF_46) |
|  | rs10519959 | 149269766 |  |  |
|  | rs6831034 | 149300138 |  |  |
|  | rs5522 | 149357475 |  |  |
|  | rs2070951 | 149358014 |  |  |
| ***NRG1***  8p12 | rs35753505 | 31474141 | NRG1 protein was originally identified as a 44-kD glycoprotein that interacts with the NEU/ERBB2 receptor tyrosine kinase to increase its phosphorylation on tyrosine residues. This protein is a signaling protein that mediates cell-cell interactions and plays critical roles in the growth and development of multiple organ systems. A variety of tissue-specifically expressed isoforms are produced from this gene through alternative promoter usage and splicing. |  |
|  | rs4733264 | 31490621 |  |  |
|  | rs6987996 | 31506771 |  |  |
|  | rs4552856 | 31555139 |  |  |
|  | rs13268778 | 31572565 |  |  |
|  | rs12679454 | 31612756 |  |  |
|  | rs16878368 | 31718367 |  |  |
|  | rs2683771 | 31746233 |  |  |
|  | rs776382 | 31758637 |  |  |
|  | rs16878644 | 31853815 |  |  |
|  | rs2068226 | 32010506 |  |  |
|  | rs6468091 | 32087606 |  |  |
|  | rs17624670 | 32126374 |  |  |
|  | rs17624997 | 32137649 |  |  |
|  | rs6468099 | 32227041 |  |  |
|  | rs16879327 | 32257621 |  |  |
|  | rs12334435 | 32393507 |  |  |
|  | rs7826312 | 32400115 |  |  |
|  | rs2466103 | 32412304 |  |  |
|  | rs2439292 | 32446882 |  |  |
|  | rs2439318 | 32527467 |  |  |
|  | rs12546380 | 32535920 |  |  |
|  | rs6468122 | 32539327 |  |  |
|  | rs10503929 | 32613983 |  |  |
|  | rs3735782 | 32624857 |  |  |
| ***SLC6A4***  17q11.2 | rs1042173 | 28525011 | Serotonin transporter protein transports the neurotransmitter serotonin from synapses to presynaptic neurons. It is an important component in the physiological response to cocaine and amphetamines. It is expressed in the central and peripheral nervous systems. | [^49-51^](#_ENREF_49) |
|  | rs3794808 | 28531793 |  |  |
|  | rs140700 | 28543389 |  |  |
|  | rs2020936 | 28550814 |  |  |
|  | rs4251417 | 28551858 |  |  |
|  | rs2020934 | 28561460 |  |  |
|  | rs2020933 | 28561755 |  |  |
| ***SYNGAP1***  6p21.3 | rs211456 | 33389381 | SYNGAP1 encodes for a protein that is a major constituent of the postsynaptic density signaling. Member of the NMDA receptor signaling complex in excitatory synapses, it may play a role in NMDA receptor-dependent control of AMPA receptor potentiation, membrane trafficking and synaptic plasticity. Regulates AMPA receptor-mediated miniature excitatory postsynaptic currents. May be involved in certain forms of brain injury, leading to long-term learning and memory deficits |  |
|  | rs413722 | 33398620 |  |  |
|  | rs9394145 | 33399778 |  |  |
|  | rs411136 | 33408542 |  |  |
|  | rs2247385 | 33421577 |  |  |
| ***TNIK***  3q26.31 | rs12486818 | 170795203 | TNIK1 encodes for a germinal centre kinase, which protein is involved in the regulation of the cytoskeleton function. TNIK protein binds DISC1 protein at synapses and regulates the composition and the function of synapses. DISC1 binding inhibits the kinase activity of TNIK leading to the degradation of several key post-synaptic density proteins, including DLG2 and modulating the surface expression of glutamate R1. The expression of TNIK in neurons is required for normal dendritic arborisation and surface expression of AMPA receptors. | [^54-57^](#_ENREF_54) |
|  | rs12488990 | 170849988 |  |  |
|  | rs6444965 | 170857240 |  |  |
|  | rs2292005 | 170885097 |  |  |
|  | rs12637875 | 170893070 |  |  |
|  | rs13098316 | 170900491 |  |  |
|  | rs7618166 | 170915666 |  |  |
|  | rs11716029 | 170927628 |  |  |
|  | rs905129 | 170952323 |  |  |
|  | rs9844004 | 170958358 |  |  |
|  | rs16856044 | 170964909 |  |  |
|  | rs7615457 | 170966507 |  |  |
|  | rs2088885 | 170971291 |  |  |
|  | rs7627954 | 170979975 |  |  |
|  | rs4894643 | 170981057 |  |  |
|  | rs4894814 | 171043762 |  |  |
|  | rs16856172 | 171067823 |  |  |
|  | rs902956 | 171103807 |  |  |

**References**

1. Baghai TC, Binder EB, Schule C, Salyakina D, Eser D, Lucae S *et al.* Polymorphisms in the angiotensin-converting enzyme gene are associated with unipolar depression, ACE activity and hypercortisolism. *Molecular Psychiatry* 2006; **11**(11)**:** 1003-1015.

2. Firouzabadi N, Shafiei M, Bahramali E, Ebrahimi SA, Bakhshandeh H, Tajik N. Association of angiotensin-converting enzyme (ACE) gene polymorphism with elevated serum ACE activity and major depression in an Iranian population. *Psychiatry Research* 2012; **200**(2)**:** 336-342.

3. Saab YB, Gard PR, Yeoman MS, Mfarrej B, El-Moalem H, Ingram MJ. Renin–angiotensin-system gene polymorphisms and depression. *Progress in Neuro-Psychopharmacology and Biological Psychiatry* 2007; **31**(5)**:** 1113-1118.

4. Turner PR, O’Connor K, Tate WP, Abraham WC. Roles of amyloid precursor protein and its fragments in regulating neural activity, plasticity and memory. *Progress in Neurobiology* 2003; **70**(1)**:** 1-32.

5. Goh WI, Lim KB, Sudhaharan T, Sem KP, Bu W, Chou AM *et al.* mDia1 and WAVE2 proteins interact directly with IRSp53 in filopodia and are involved in filopodium formation. *Journal of Biological Chemistry* 2012; **287**(7)**:** 4702-4714.

6. Kim MH, Choi J, Yang J, Chung W, Kim JH, Paik SK *et al.* Enhanced NMDA receptor-mediated synaptic transmission, enhanced long-term potentiation, and impaired learning and memory in mice lacking IRSp53. *Journal of Neuroscience* 2009; **29**(5)**:** 1586-1595.

7. Chen L, Lawlor DA, Lewis SJ, Yuan W, Abdollahi MR, Timpson NJ *et al.* Genetic association study of BDNF in depression: finding from two cohort studies and a meta-analysis. *American Journal of Medical Genetics Part B Neuropsychiatric Genetics* 2008; **147B**(6)**:** 814-821.

8. Frustaci A, Pozzi G, Gianfagna F, Manzoli L, Boccia S. Meta-analysis of the brain-derived neurotrophic factor gene (BDNF) Val66Met polymorphism in anxiety disorders and anxiety-related personality traits. *Neuropsychobiology* 2008; **58**(3-4)**:** 163-170.

9. Gratacos M, Gonzalez JR, Mercader JM, de Cid R, Urretavizcaya M, Estivill X. Brain-derived neurotrophic factor Val66Met and psychiatric disorders: meta-analysis of case-control studies confirm association to substance-related disorders, eating disorders, and schizophrenia. *Biological Psychiatry* 2007; **61**(7)**:** 911-922.

10. Verhagen M, Van Der Meij A, van Deurzen P, Janzing J, Arias-Vasquez A, Buitelaar J *et al.* Meta-analysis of the BDNF Val66Met polymorphism in major depressive disorder: effects of gender and ethnicity. *Molecular Psychiatry* 2008; **15**(3)**:** 260-271.

11. Biederer T, Sara Y, Mozhayeva M, Atasoy D, Liu X, Kavalali ET *et al.* SynCAM, a synaptic adhesion molecule that drives synapse assembly. *Science* 2002; **297**(5586)**:** 1525-1531.

12. Boles KS, Barchet W, Diacovo T, Cella M, Colonna M. The tumor suppressor TSLC1/NECL-2 triggers NK-cell and CD8+ T-cell responses through the cell-surface receptor CRTAM. *Blood* 2005; **106**(3)**:** 779-786.

13. Hollins F, Kaur D, Yang W, Cruse G, Saunders R, Sutcliffe A *et al.* Human airway smooth muscle promotes human lung mast cell survival, proliferation, and constitutive activation: cooperative roles for CADM1, stem cell factor, and IL-6. *Journal of Immunology* 2008; **181**(4)**:** 2772-2780.

14. Raveendran R, Devi Suma Priya S, Mayadevi M, Steephan M, Santhoshkumar TR, Cheriyan J *et al.* Phosphorylation status of the NR2B subunit of NMDA receptor regulates its interaction with calcium/calmodulin-dependent protein kinase II. *J Neurochem* 2009; **110**(1)**:** 92-105.

15. Drabant EM, Hariri AR, Meyer-Lindenberg A, Munoz KE, Mattay VS, Kolachana BS *et al.* Catechol O-methyltransferase val158met genotype and neural mechanisms related to affective arousal and regulation. *Archives of General Psychiatry* 2006; **63**(12)**:** 1396.

16. Hettema JM, An SS, Bukszar J, van den Oord EJ, Neale MC, Kendler KS *et al.* Catechol-O-methyltransferase contributes to genetic susceptibility shared among anxiety spectrum phenotypes. *Biological Psychiatry* 2008; **64**(4)**:** 302-310.

17. Massat I, Souery D, Del-Favero J, Nothen M, Blackwood D, Muir W *et al.* Association between COMT (Val158Met) functional polymorphism and early onset in patients with major depressive disorder in a European multicenter genetic association study. *Molecular Psychiatry* 2004; **10**(6)**:** 598-605.

18. Gomez L, Wigg K, Feng Y, Kiss E, Kapornai K, Tamás Z *et al.* G72/G30 (DAOA) and juvenile‐onset mood disorders. *American Journal of Medical Genetics Part B Neuropsychiatric Genetics* 2009; **150**(7)**:** 1007-1012.

19. Rietschel M, Beckmann L, Strohmaier J, Georgi A, Karpushova A, Schirmbeck F *et al.* G72 and its association with major depression and neuroticism in large population-based groups from Germany. *AmJPsychiatry* 2008; **165**(6)**:** 753-762.

20. Williams NM, Green EK, Macgregor S, Dwyer S, Norton N, Williams H *et al.* Variation at the DAOA/G30 locus influences susceptibility to major mood episodes but not psychosis in schizophrenia and bipolar disorder. *Archives of General Psychiatry* 2006; **63**(4)**:** 366.

21. Hashimoto R, Numakawa T, Ohnishi T, Kumamaru E, Yagasaki Y, Ishimoto T *et al.* Impact of the DISC1 Ser704Cys polymorphism on risk for major depression, brain morphology and ERK signaling. *Human Molecular Genetics* 2006; **15**(20)**:** 3024-3033.

22. Okuda A, Kishi T, Okochi T, Ikeda M, Kitajima T, Tsunoka T *et al.* Translin-Associated Factor X Gene (TSNAX) may be Associated with Female major Depressive Disorder in the Japanese Population. *Neuromolecular Medicine* 2009.

23. Schosser A, Cohen‐Woods S, Gaysina D, Chow PC, Martucci L, Farmer A *et al.* NRG1 gene in recurrent major depression: No association in a large‐scale case–control association study. *American Journal of Medical Genetics Part B Neuropsychiatric Genetics* 2010; **153**(1)**:** 141-147.

24. Craven SE, El-Husseini AE, Bredt DS. Synaptic targeting of the postsynaptic density protein PSD-95 mediated by lipid and protein motifs. *Neuron* 1999; **22**(3)**:** 497-509.

25. Tao YX, Rumbaugh G, Wang GD, Petralia RS, Zhao C, Kauer FW *et al.* Impaired NMDA receptor-mediated postsynaptic function and blunted NMDA receptor-dependent persistent pain in mice lacking postsynaptic density-93 protein. *Journal of Neurosciece* 2003; **23**(17)**:** 6703-6712.

26. Fan J, Vasuta OC, Zhang LY, Wang L, George A, Raymond LA. N-methyl-D-aspartate receptor subunit- and neuronal-type dependence of excitotoxic signaling through post-synaptic density 95. *Journal of Neurochemistry* 2010; **115**(4)**:** 1045-1056.

27. Chen X, Nelson CD, Li X, Winters CA, Azzam R, Sousa AA *et al.* PSD-95 is required to sustain the molecular organization of the postsynaptic density. *Journal of Neuroscience* 2011; **31**(17)**:** 6329-6338.

28. Takeuchi M, Hata Y, Hirao K, Toyoda A, Irie M, Takai Y. SAPAPs. A family of PSD-95/SAP90-associated proteins localized at postsynaptic density. *Journal of Biological Chemistry* 1997; **272**(18)**:** 11943-11951.

29. Hirao K, Hata Y, Ide N, Takeuchi M, Irie M, Yao I *et al.* A novel multiple PDZ domain-containing molecule interacting with N-methyl-D-aspartate receptors and neuronal cell adhesion proteins. *Journal of Biological Chemistry* 1998; **273**(33)**:** 21105-21110.

30. Husi H, Ward MA, Choudhary JS, Blackstock WP, Grant SG. Proteomic analysis of NMDA receptor-adhesion protein signaling complexes. *Nature Neuroscience* 2000; **3**(7)**:** 661-669.

31. Binder EB, Salyakina D, Lichtner P, Wochnik GM, Ising M, Putz B *et al.* Polymorphisms in FKBP5 are associated with increased recurrence of depressive episodes and rapid response to antidepressant treatment. *Nature Genetics* 2004; **36**(12)**:** 1319-1325.

32. Gawlik M, Moller-Ehrlich K, Mende M, Jovnerovski M, Jung S, Jabs B *et al.* Is FKBP5 a genetic marker of affective psychosis? A case control study and analysis of disease related traits. *BMC Psychiatry* 2006; **6:** 52.

33. Willour VL, Chen H, Toolan J, Belmonte P, Cutler DJ, Goes FS *et al.* Family-based association of FKBP5 in bipolar disorder. *Molecular Psychiatry* 2009; **14**(3)**:** 261-268.

34. Hiskens R, Vatish M, Hill C, Davey J, Ladds G. Specific in vivo binding of activator of G protein signalling 1 to the Gbeta1 subunit. *Biochem Biophys Res Commun* 2005; **337**(4)**:** 1038-1046.

35. Chen YC, Kung SS, Chen BY, Hung CC, Chen CC, Wang TY *et al.* Identifications, classification, and evolution of the vertebrate alpha-amino-3-hydroxy-5-methyl-4-isoxazole propionic acid (AMPA) receptor subunit genes. *Journal of Molecular Evolution* 2001; **53**(6)**:** 690-702.

36. Ali F, Meier R. Primate home range and GRIN2A, a receptor gene involved in neuronal plasticity: implications for the evolution of spatial memory. *Genes Brain Behav* 2009; **8**(4)**:** 435-441.

37. Abdolmaleky HM, Thiagalingam S, Wilcox M. Genetics and epigenetics in major psychiatric disorders: dilemmas, achievements, applications, and future scope. *American Journal of Pharmacogenomics* 2005; **5**(3)**:** 149-160.

38. Harrison PJ, Lyon L, Sartorius LJ, Burnet PW, Lane TA. The group II metabotropic glutamate receptor 3 (mGluR3, mGlu3, GRM3): expression, function and involvement in schizophrenia. *Journal of Psychopharmacology* 2008; **22**(3)**:** 308-322.

39. Corti C, Xuereb JH, Corsi M, Ferraguti F. Identification and characterization of the promoter region of the GRM3 gene. *Biochemical and Biophysical Reseacrh Communications* 2001; **286**(2)**:** 381-387.

40. Fukaya M, Kamata A, Hara Y, Tamaki H, Katsumata O, Ito N *et al.* SynArfGEF is a guanine nucleotide exchange factor for Arf6 and localizes preferentially at post-synaptic specializations of inhibitory synapses. *Journal of Neurochemistry* 2011; **116**(6)**:** 1122-1137.

41. Carlisle HJ, Luong TN, Medina-Marino A, Schenker L, Khorosheva E, Indersmitten T *et al.* Deletion of densin-180 results in abnormal behaviors associated with mental illness and reduces mGluR5 and DISC1 in the postsynaptic density fraction. *Journal of Neuroscience* 2011; **31**(45)**:** 16194-16207.

42. Bet PM, Penninx BW, Bochdanovits Z, Uitterlinden AG, Beekman AT, van Schoor NM *et al.* Glucocorticoid receptor gene polymorphisms and childhood adversity are associated with depression: New evidence for a gene-environment interaction. *American Journal of Medical Genetics B Neuropsychiatric Genetics* 2009; **150B**(5)**:** 660-669.

43. van Rossum EF, Binder EB, Majer M, Koper JW, Ising M, Modell S *et al.* Polymorphisms of the glucocorticoid receptor gene and major depression. *Biological Psychiatry* 2006; **59**(8)**:** 681-688.

44. van West D, Van Den EF, Del Favero J, Souery D, Norrback KF, Van Duijn C *et al.* Glucocorticoid receptor gene-based SNP analysis in patients with recurrent major depression. *Neuropsychopharmacology* 2006; **31**(3)**:** 620-627.

45. Zobel A, Jessen F, von Widdern O, Schuhmacher A, Hofels S, Metten M *et al.* Unipolar depression and hippocampal volume: impact of DNA sequence variants of the glucocorticoid receptor gene. *American Journal of Medical Genetics Part B Neuropsychiatric Genetics* 2008; **147B**(6)**:** 836-843.

46. Kuningas M, de Rijk RH, Westendorp RG, Jolles J, Slagboom PE, van Heemst D. Mental performance in old age dependent on cortisol and genetic variance in the mineralocorticoid and glucocorticoid receptors. *Neuropsychopharmacology* 2007; **32**(6)**:** 1295-1301.

47. Green EK, Raybould R, Macgregor S, Gordon-Smith K, Heron J, Hyde S *et al.* Operation of the schizophrenia susceptibility gene, neuregulin 1, across traditional diagnostic boundaries to increase risk for bipolar disorder. *Archives of General Psychiatry* 2005; **62**(6)**:** 642.

48. Thomson P, Christoforou A, Morris S, Adie E, Pickard B, Porteous D *et al.* Association of Neuregulin 1 with schizophrenia and bipolar disorder in a second cohort from the Scottish population. *Molecular Psychiatry* 2006; **12**(1)**:** 94-104.

49. Clarke TK, Treutlein J, Zimmermann US, Kiefer F, Skowronek MH, Rietschel M *et al.* HPA-axis activity in alcoholism: examples for a gene-environment interaction. *Addiction Biology* 2008; **13**(1)**:** 1-14.

50. Karg K, Burmeister M, Shedden K, Sen S. The serotonin transporter promoter variant (5-HTTLPR), stress, and depression meta-analysis revisited: evidence of genetic moderation. *Archives of General Psychiatry* 2011; **68**(5)**:** 444.

51. Wray NR, James MR, Gordon SD, Dumenil T, Ryan L, Coventry WL *et al.* Accurate, Large-Scale Genotyping of 5HTTLPR and Flanking Single Nucleotide Polymorphisms in an Association Study of Depression, Anxiety, and Personality Measures. *Biological Psychiatry* 2009; **66**(5)**:** 468-476.

52. Clement JP, Aceti M, Creson TK, Ozkan ED, Shi Y, Reish NJ *et al.* Pathogenic SYNGAP1 mutations impair cognitive development by disrupting maturation of dendritic spine synapses. *Cell* 2012; **151**(4)**:** 709-723.

53. Krapivinsky G, Medina I, Krapivinsky L, Gapon S, Clapham DE. SynGAP-MUPP1-CaMKII synaptic complexes regulate p38 MAP kinase activity and NMDA receptor-dependent synaptic AMPA receptor potentiation. *Neuron* 2004; **43**(4)**:** 563-574.

54. Gui J, Yang B, Wu J, Zhou X. The enormous influence of TNIK knockdown on intracellular signals and cell survival. *Hum Cell* 2011; **24**(3)**:** 121-126.

55. Potkin SG, Turner JA, Guffanti G, Lakatos A, Fallon JH, Nguyen DD *et al.* A genome-wide association study of schizophrenia using brain activation as a quantitative phenotype. *Schizophr Bull* 2009; **35**(1)**:** 96-108.

56. Wang Q, Charych EI, Pulito VL, Lee JB, Graziane NM, Crozier RA *et al.* The psychiatric disease risk factors DISC1 and TNIK interact to regulate synapse composition and function. *Mol Psychiatry* 2011; **16**(10)**:** 1006-1023.

57. Hussain NK, Hsin H, Huganir RL, Sheng M. MINK and TNIK differentially act on Rap2-mediated signal transduction to regulate neuronal structure and AMPA receptor function. *J Neurosci* 2010; **30**(44)**:** 14786-14794.
