# Supplementary material for: Psychometric precision in phenotype definition is a useful step in molecular genetic investigation of psychiatric disorders
Source: Transl Psychiatry. 2015 Jun 30;5(6):e593–. doi: 10.1038/tp.2015.86 (PMC4490295; doi:10.1038/tp.2015.86)
Supplement: Supplementary Table 3 [file tp201586x3.doc]

| Table S3 SNP association results based on bi-factor method | | | | | | |  |  |  |  |  |  |  |  |  |  |  |
| --- | --- | --- | --- | --- | --- | --- | --- | --- | --- | --- | --- | --- | --- | --- | --- | --- | --- |
|  |  |  | Global affective symptoms | | | Anxiety symptoms | | | Depression symptoms | | | Social dysfunction | | | Somatic symptoms | | |
| Gene | SNP |  | beta coef | p value | effect size | beta coef | p value | effect size | beta coef | p value | effect size | beta coef | p value | effect size | beta coef | p value | effect size |
| ACE | rs1800764 |  | -0.071 | 0.079 | 0.005 | 0.135 | 0.094 | 0.018 | 0.136 | 0.026 | 0.018 | 0.017 | 0.693 | 0.000 | -0.013 | 0.775 | 0.000 |
| ACE | rs4291 |  | -0.053 | 0.181 | 0.003 | 0.111 | 0.150 | 0.012 | 0.097 | 0.097 | 0.009 | 0.019 | 0.663 | 0.000 | 0.007 | 0.876 | 0.000 |
| ACE | rs4305 |  | -0.069 | 0.088 | 0.005 | 0.125 | 0.118 | 0.016 | 0.107 | 0.078 | 0.011 | 0.008 | 0.849 | 0.000 | -0.005 | 0.914 | 0.000 |
| ACE | rs4324 |  | 0.098 | 0.014 | 0.010 | -0.132 | 0.091 | 0.017 | -0.140 | 0.015 | 0.020 | -0.060 | 0.185 | 0.004 | -0.013 | 0.780 | 0.000 |
| ACE | rs4353 |  | 0.105 | 0.008 | 0.011 | -0.122 | 0.117 | 0.015 | -0.117 | 0.042 | 0.014 | -0.063 | 0.157 | 0.004 | -0.029 | 0.539 | 0.001 |
| ACE | rs4461142 |  | -0.059 | 0.164 | 0.003 | 0.079 | 0.334 | 0.006 | 0.064 | 0.311 | 0.004 | 0.021 | 0.654 | 0.000 | -0.030 | 0.554 | 0.001 |
| ACE | rs4267385 |  | 0.089 | 0.022 | 0.008 | -0.092 | 0.231 | 0.008 | -0.089 | 0.102 | 0.008 | -0.043 | 0.310 | 0.002 | 0.016 | 0.708 | 0.000 |
| ACE | rs496859 |  | 0.046 | 0.247 | 0.002 | -0.134 | 0.094 | 0.018 | -0.112 | 0.060 | 0.013 | -0.114 | 0.011 | 0.013 | -0.091 | 0.041 | 0.008 |
| APP | rs7277612 |  | -0.066 | 0.098 | 0.004 | 0.039 | 0.604 | 0.002 | 0.065 | 0.273 | 0.004 | 0.039 | 0.388 | 0.002 | 0.007 | 0.880 | 0.000 |
| APP | rs17001492 |  | -0.022 | 0.601 | 0.000 | 0.057 | 0.491 | 0.003 | -0.034 | 0.608 | 0.001 | -0.039 | 0.378 | 0.002 | -0.007 | 0.895 | 0.000 |
| APP | rs2829981 |  | 0.027 | 0.500 | 0.001 | -0.011 | 0.884 | 0.000 | -0.065 | 0.276 | 0.004 | -0.088 | 0.046 | 0.008 | -0.026 | 0.599 | 0.001 |
| APP | rs4817076 |  | 0.008 | 0.826 | 0.000 | 0.016 | 0.823 | 0.000 | -0.022 | 0.713 | 0.000 | -0.039 | 0.382 | 0.002 | -0.022 | 0.639 | 0.000 |
| APP | rs2830012 |  | -0.041 | 0.298 | 0.002 | 0.099 | 0.196 | 0.010 | 0.051 | 0.405 | 0.003 | 0.012 | 0.788 | 0.000 | 0.015 | 0.748 | 0.000 |
| APP | rs3787644 |  | -0.021 | 0.591 | 0.000 | 0.017 | 0.808 | 0.000 | 0.057 | 0.334 | 0.003 | 0.022 | 0.604 | 0.000 | 0.009 | 0.847 | 0.000 |
| APP | rs2830034 |  | 0.009 | 0.827 | 0.000 | -0.069 | 0.354 | 0.005 | -0.006 | 0.921 | 0.000 | -0.037 | 0.421 | 0.001 | 0.012 | 0.794 | 0.000 |
| BAIAP2 | rs4969361 |  | -0.025 | 0.509 | 0.001 | 0.092 | 0.212 | 0.008 | 0.058 | 0.315 | 0.003 | 0.075 | 0.081 | 0.006 | 0.008 | 0.847 | 0.000 |
| BAIAP2 | rs8080252 |  | 0.047 | 0.218 | 0.002 | -0.053 | 0.468 | 0.003 | -0.022 | 0.704 | 0.000 | -0.093 | 0.044 | 0.009 | -0.028 | 0.563 | 0.001 |
| BAIAP2 | rs12937996 |  | -0.057 | 0.152 | 0.003 | -0.017 | 0.828 | 0.000 | 0.050 | 0.383 | 0.003 | 0.008 | 0.860 | 0.000 | 0.042 | 0.391 | 0.002 |
| BAIAP2 | rs4969245 |  | -0.102 | 0.013 | 0.010 | 0.157 | 0.050 | 0.025 | 0.045 | 0.439 | 0.002 | 0.049 | 0.308 | 0.002 | 0.086 | 0.071 | 0.007 |
| BAIAP2 | rs12944983 |  | 0.021 | 0.591 | 0.000 | -0.006 | 0.938 | 0.000 | 0.033 | 0.561 | 0.001 | -0.012 | 0.794 | 0.000 | -0.047 | 0.286 | 0.002 |
| BDNF | rs6265 |  | 0.003 | 0.930 | 0.000 | -0.028 | 0.700 | 0.001 | -0.047 | 0.426 | 0.002 | 0.013 | 0.773 | 0.000 | 0.029 | 0.535 | 0.001 |
| BDNF | rs11030101 |  | 0.009 | 0.824 | 0.000 | -0.084 | 0.274 | 0.007 | 0.009 | 0.885 | 0.000 | -0.020 | 0.667 | 0.000 | -0.017 | 0.731 | 0.000 |
| BDNF | rs11030107 |  | -0.028 | 0.478 | 0.001 | 0.102 | 0.167 | 0.010 | 0.043 | 0.467 | 0.002 | 0.035 | 0.455 | 0.001 | 0.029 | 0.522 | 0.001 |
| BDNF | rs7103411 |  | 0.017 | 0.655 | 0.000 | -0.018 | 0.808 | 0.000 | -0.009 | 0.881 | 0.000 | -0.006 | 0.893 | 0.000 | 0.005 | 0.907 | 0.000 |
| BDNF | rs7127507 |  | -0.035 | 0.389 | 0.001 | 0.101 | 0.194 | 0.010 | 0.009 | 0.884 | 0.000 | 0.026 | 0.584 | 0.001 | 0.021 | 0.664 | 0.000 |
| CADM1 | rs1048932 |  | -0.015 | 0.701 | 0.000 | 0.074 | 0.329 | 0.005 | 0.026 | 0.646 | 0.001 | 0.032 | 0.484 | 0.001 | 0.088 | 0.055 | 0.008 |
| CADM1 | rs17118125 |  | 0.002 | 0.950 | 0.000 | 0.046 | 0.541 | 0.002 | 0.099 | 0.092 | 0.010 | 0.049 | 0.268 | 0.002 | 0.058 | 0.203 | 0.003 |
| CADM1 | rs10502199 |  | -0.030 | 0.430 | 0.001 | 0.089 | 0.227 | 0.008 | -0.040 | 0.494 | 0.002 | -0.035 | 0.465 | 0.001 | 0.023 | 0.619 | 0.001 |
| CAMK2A | rs2241695 |  | 0.020 | 0.625 | 0.000 | 0.032 | 0.684 | 0.001 | -0.010 | 0.882 | 0.000 | -0.065 | 0.155 | 0.004 | -0.037 | 0.438 | 0.001 |
| CAMK2A | rs4958469 |  | -0.020 | 0.608 | 0.000 | 0.046 | 0.516 | 0.002 | -0.014 | 0.800 | 0.000 | -0.015 | 0.738 | 0.000 | -0.012 | 0.809 | 0.000 |
| CAMK2A | rs2053053 |  | 0.020 | 0.601 | 0.000 | -0.006 | 0.933 | 0.000 | -0.063 | 0.289 | 0.004 | -0.056 | 0.194 | 0.003 | -0.044 | 0.344 | 0.002 |
| CAMK2A | rs3756578 |  | 0.007 | 0.862 | 0.000 | 0.039 | 0.601 | 0.002 | -0.029 | 0.627 | 0.001 | -0.062 | 0.162 | 0.004 | -0.042 | 0.363 | 0.002 |
| CAMK2A | rs7711562 |  | -0.011 | 0.791 | 0.000 | -0.005 | 0.943 | 0.000 | -0.054 | 0.393 | 0.003 | -0.024 | 0.600 | 0.001 | -0.040 | 0.406 | 0.002 |
| CAMK2A | rs2288799 |  | -0.018 | 0.637 | 0.000 | -0.010 | 0.887 | 0.000 | -0.134 | 0.021 | 0.018 | -0.061 | 0.156 | 0.004 | -0.086 | 0.061 | 0.007 |
| CAMK2A | rs4958445 |  | -0.026 | 0.496 | 0.001 | 0.033 | 0.657 | 0.001 | -0.052 | 0.376 | 0.003 | -0.042 | 0.333 | 0.002 | -0.005 | 0.919 | 0.000 |
| CAMK2A | rs4958902 |  | -0.026 | 0.532 | 0.001 | 0.017 | 0.831 | 0.000 | -0.016 | 0.794 | 0.000 | -0.053 | 0.246 | 0.003 | -0.009 | 0.857 | 0.000 |
| CAMK2A | rs919740 |  | 0.034 | 0.379 | 0.001 | -0.122 | 0.116 | 0.015 | -0.126 | 0.050 | 0.016 | 0.003 | 0.952 | 0.000 | 0.008 | 0.872 | 0.000 |
| CAMK2A | rs11167499 |  | 0.031 | 0.423 | 0.001 | -0.062 | 0.387 | 0.004 | 0.036 | 0.532 | 0.001 | -0.012 | 0.772 | 0.000 | -0.034 | 0.426 | 0.001 |
| CAMK2A | rs2295223 |  | 0.020 | 0.596 | 0.000 | 0.094 | 0.182 | 0.009 | -0.052 | 0.366 | 0.003 | -0.001 | 0.991 | 0.000 | 0.085 | 0.087 | 0.007 |
| COMT | rs7289747 |  | 0.008 | 0.838 | 0.000 | -0.008 | 0.904 | 0.000 | 0.059 | 0.294 | 0.003 | -0.030 | 0.449 | 0.001 | 0.007 | 0.865 | 0.000 |
| COMT | rs737865 |  | -0.015 | 0.698 | 0.000 | -0.031 | 0.674 | 0.001 | -0.048 | 0.411 | 0.002 | -0.015 | 0.726 | 0.000 | 0.034 | 0.478 | 0.001 |
| COMT | rs6269 |  | -0.050 | 0.181 | 0.003 | 0.064 | 0.375 | 0.004 | 0.001 | 0.981 | 0.000 | -0.024 | 0.587 | 0.001 | 0.086 | 0.053 | 0.007 |
| COMT | rs4633 |  | -0.041 | 0.267 | 0.002 | 0.011 | 0.881 | 0.000 | -0.037 | 0.534 | 0.001 | -0.008 | 0.855 | 0.000 | 0.046 | 0.290 | 0.002 |
| COMT | rs4818 |  | -0.044 | 0.243 | 0.002 | 0.071 | 0.324 | 0.005 | 0.006 | 0.928 | 0.000 | -0.023 | 0.597 | 0.001 | 0.083 | 0.063 | 0.007 |
| COMT | rs4680 |  | -0.036 | 0.341 | 0.001 | 0.023 | 0.756 | 0.001 | -0.039 | 0.515 | 0.002 | -0.017 | 0.699 | 0.000 | 0.037 | 0.393 | 0.001 |
| COMT | rs165599 |  | 0.018 | 0.647 | 0.000 | 0.000 | 0.995 | 0.000 | -0.050 | 0.383 | 0.003 | -0.002 | 0.955 | 0.000 | 0.030 | 0.481 | 0.001 |
| DAOA | rs778294 |  | 0.034 | 0.378 | 0.001 | -0.161 | 0.035 | 0.026 | 0.001 | 0.979 | 0.000 | -0.082 | 0.061 | 0.007 | -0.095 | 0.035 | 0.009 |
| DAOA | rs3918342 |  | 0.000 | 0.996 | 0.000 | 0.011 | 0.883 | 0.000 | 0.026 | 0.655 | 0.001 | -0.021 | 0.642 | 0.000 | 0.022 | 0.648 | 0.000 |
| DAOA | rs1421292 |  | -0.011 | 0.781 | 0.000 | 0.038 | 0.604 | 0.001 | 0.044 | 0.432 | 0.002 | -0.003 | 0.937 | 0.000 | 0.028 | 0.554 | 0.001 |
| DISC1 | rs1059595 |  | 0.038 | 0.328 | 0.001 | -0.064 | 0.390 | 0.004 | 0.019 | 0.743 | 0.000 | -0.048 | 0.306 | 0.002 | -0.046 | 0.342 | 0.002 |
| DISC1 | rs2570409 |  | 0.043 | 0.253 | 0.002 | 0.002 | 0.983 | 0.000 | 0.040 | 0.472 | 0.002 | 0.008 | 0.847 | 0.000 | -0.024 | 0.588 | 0.001 |
| DISC1 | rs3738401 |  | 0.039 | 0.312 | 0.002 | 0.023 | 0.743 | 0.001 | -0.075 | 0.209 | 0.006 | -0.038 | 0.383 | 0.001 | -0.035 | 0.429 | 0.001 |
| DISC1 | rs1934909 |  | 0.021 | 0.579 | 0.000 | -0.052 | 0.471 | 0.003 | 0.081 | 0.128 | 0.007 | -0.026 | 0.532 | 0.001 | -0.061 | 0.191 | 0.004 |
| DISC1 | rs12027635 |  | 0.035 | 0.355 | 0.001 | 0.067 | 0.328 | 0.004 | -0.108 | 0.077 | 0.012 | 0.053 | 0.209 | 0.003 | 0.066 | 0.118 | 0.004 |
| DISC1 | rs2793085 |  | -0.106 | 0.005 | 0.011 | 0.147 | 0.055 | 0.022 | 0.084 | 0.157 | 0.007 | -0.013 | 0.760 | 0.000 | -0.012 | 0.799 | 0.000 |
| DISC1 | rs1322783 |  | 0.015 | 0.703 | 0.000 | -0.143 | 0.061 | 0.020 | 0.026 | 0.660 | 0.001 | -0.081 | 0.050 | 0.007 | -0.090 | 0.044 | 0.008 |
| DISC1 | rs6675281 |  | 0.015 | 0.691 | 0.000 | -0.050 | 0.506 | 0.003 | 0.056 | 0.326 | 0.003 | -0.014 | 0.748 | 0.000 | -0.083 | 0.059 | 0.007 |
| DISC1 | rs9431708 |  | -0.027 | 0.501 | 0.001 | 0.186 | 0.017 | 0.035 | 0.058 | 0.352 | 0.003 | -0.014 | 0.753 | 0.000 | 0.056 | 0.216 | 0.003 |
| DISC1 | rs7541019 |  | 0.025 | 0.504 | 0.001 | -0.070 | 0.319 | 0.005 | -0.032 | 0.576 | 0.001 | -0.007 | 0.871 | 0.000 | -0.071 | 0.117 | 0.005 |
| DISC1 | rs6670775 |  | 0.018 | 0.640 | 0.000 | -0.066 | 0.367 | 0.004 | -0.073 | 0.210 | 0.005 | 0.044 | 0.325 | 0.002 | -0.019 | 0.677 | 0.000 |
| DISC1 | rs821597 |  | 0.029 | 0.470 | 0.001 | -0.020 | 0.784 | 0.000 | -0.036 | 0.545 | 0.001 | 0.046 | 0.301 | 0.002 | 0.012 | 0.790 | 0.000 |
| DISC1 | rs821616 |  | 0.050 | 0.216 | 0.003 | -0.031 | 0.674 | 0.001 | -0.021 | 0.717 | 0.000 | 0.045 | 0.297 | 0.002 | 0.012 | 0.803 | 0.000 |
| DISC1 | rs11802446 |  | 0.056 | 0.150 | 0.003 | 0.004 | 0.955 | 0.000 | 0.017 | 0.762 | 0.000 | -0.021 | 0.646 | 0.000 | -0.042 | 0.365 | 0.002 |
| DLG2 | rs891773 |  | -0.036 | 0.346 | 0.001 | 0.030 | 0.686 | 0.001 | 0.022 | 0.715 | 0.000 | 0.084 | 0.054 | 0.007 | 0.035 | 0.426 | 0.001 |
| DLG2 | rs11233640 |  | 0.033 | 0.399 | 0.001 | 0.027 | 0.737 | 0.001 | -0.055 | 0.378 | 0.003 | -0.119 | 0.009 | 0.014 | -0.011 | 0.808 | 0.000 |
| DLG2 | rs11233649 |  | 0.022 | 0.569 | 0.000 | -0.012 | 0.869 | 0.000 | -0.017 | 0.775 | 0.000 | 0.011 | 0.798 | 0.000 | -0.047 | 0.300 | 0.002 |
| DLG2 | rs3815988 |  | 0.017 | 0.662 | 0.000 | -0.027 | 0.707 | 0.001 | -0.015 | 0.791 | 0.000 | 0.005 | 0.913 | 0.000 | -0.033 | 0.470 | 0.001 |
| DLG2 | rs11233660 |  | 0.005 | 0.889 | 0.000 | 0.111 | 0.111 | 0.012 | 0.022 | 0.696 | 0.000 | 0.031 | 0.419 | 0.001 | -0.007 | 0.859 | 0.000 |
| DLG2 | rs6592123 |  | 0.013 | 0.739 | 0.000 | -0.042 | 0.559 | 0.002 | -0.021 | 0.718 | 0.000 | -0.002 | 0.958 | 0.000 | -0.034 | 0.453 | 0.001 |
| DLG2 | rs6592124 |  | 0.009 | 0.818 | 0.000 | -0.046 | 0.520 | 0.002 | -0.013 | 0.815 | 0.000 | 0.001 | 0.975 | 0.000 | -0.032 | 0.475 | 0.001 |
| DLG2 | rs485199 |  | -0.002 | 0.954 | 0.000 | -0.050 | 0.503 | 0.003 | 0.033 | 0.570 | 0.001 | 0.043 | 0.329 | 0.002 | 0.003 | 0.949 | 0.000 |
| DLG2 | rs7945102 |  | 0.025 | 0.533 | 0.001 | -0.086 | 0.271 | 0.007 | -0.098 | 0.091 | 0.010 | -0.091 | 0.044 | 0.008 | -0.026 | 0.572 | 0.001 |
| DLG2 | rs7126807 |  | -0.030 | 0.444 | 0.001 | -0.055 | 0.461 | 0.003 | -0.008 | 0.899 | 0.000 | -0.015 | 0.747 | 0.000 | -0.003 | 0.958 | 0.000 |
| DLG2 | rs1037353 |  | -0.030 | 0.444 | 0.001 | -0.055 | 0.461 | 0.003 | -0.008 | 0.899 | 0.000 | -0.015 | 0.747 | 0.000 | -0.003 | 0.958 | 0.000 |
| DLG2 | rs1400313 |  | 0.028 | 0.475 | 0.001 | -0.037 | 0.614 | 0.001 | -0.045 | 0.437 | 0.002 | 0.025 | 0.573 | 0.001 | -0.022 | 0.641 | 0.000 |
| DLG2 | rs4480568 |  | -0.063 | 0.115 | 0.004 | 0.143 | 0.066 | 0.020 | 0.029 | 0.613 | 0.001 | -0.005 | 0.906 | 0.000 | 0.059 | 0.213 | 0.003 |
| DLG2 | rs7926468 |  | 0.041 | 0.280 | 0.002 | -0.153 | 0.041 | 0.023 | 0.013 | 0.818 | 0.000 | -0.062 | 0.166 | 0.004 | -0.062 | 0.197 | 0.004 |
| DLG2 | rs10792694 |  | 0.023 | 0.546 | 0.001 | -0.019 | 0.796 | 0.000 | -0.039 | 0.497 | 0.002 | 0.046 | 0.303 | 0.002 | -0.010 | 0.817 | 0.000 |
| DLG2 | rs10898159 |  | -0.020 | 0.589 | 0.000 | -0.055 | 0.427 | 0.003 | 0.007 | 0.890 | 0.000 | 0.011 | 0.793 | 0.000 | 0.013 | 0.758 | 0.000 |
| DLG2 | rs10898161 |  | 0.030 | 0.414 | 0.001 | 0.033 | 0.636 | 0.001 | -0.079 | 0.136 | 0.006 | -0.042 | 0.295 | 0.002 | -0.066 | 0.146 | 0.004 |
| DLG2 | rs1483408 |  | -0.025 | 0.543 | 0.001 | 0.056 | 0.460 | 0.003 | -0.016 | 0.784 | 0.000 | -0.069 | 0.112 | 0.005 | -0.012 | 0.801 | 0.000 |
| DLG2 | rs7125294 |  | 0.000 | 0.997 | 0.000 | 0.058 | 0.432 | 0.003 | -0.013 | 0.820 | 0.000 | -0.002 | 0.973 | 0.000 | -0.023 | 0.629 | 0.001 |
| DLG2 | rs11824888 |  | -0.062 | 0.117 | 0.004 | 0.092 | 0.256 | 0.008 | -0.015 | 0.795 | 0.000 | 0.013 | 0.771 | 0.000 | 0.027 | 0.585 | 0.001 |
| DLG2 | rs7129994 |  | -0.020 | 0.618 | 0.000 | -0.033 | 0.644 | 0.001 | -0.084 | 0.131 | 0.007 | 0.051 | 0.259 | 0.003 | -0.007 | 0.882 | 0.000 |
| DLG2 | rs11234131 |  | 0.033 | 0.394 | 0.001 | -0.002 | 0.976 | 0.000 | -0.024 | 0.671 | 0.001 | -0.039 | 0.405 | 0.002 | 0.047 | 0.265 | 0.002 |
| DLG2 | rs548098 |  | -0.059 | 0.135 | 0.003 | 0.132 | 0.079 | 0.017 | 0.063 | 0.297 | 0.004 | -0.027 | 0.539 | 0.001 | 0.025 | 0.570 | 0.001 |
| DLG2 | rs11234161 |  | 0.019 | 0.623 | 0.000 | -0.010 | 0.883 | 0.000 | -0.027 | 0.635 | 0.001 | -0.025 | 0.588 | 0.001 | 0.037 | 0.352 | 0.001 |
| DLG2 | rs10898305 |  | -0.048 | 0.205 | 0.002 | -0.010 | 0.884 | 0.000 | -0.044 | 0.404 | 0.002 | -0.030 | 0.485 | 0.001 | 0.038 | 0.377 | 0.001 |
| DLG2 | rs1943711 |  | 0.017 | 0.665 | 0.000 | -0.047 | 0.517 | 0.002 | 0.022 | 0.697 | 0.000 | 0.002 | 0.971 | 0.000 | 0.035 | 0.414 | 0.001 |
| DLG2 | rs1943733 |  | 0.057 | 0.148 | 0.003 | -0.143 | 0.070 | 0.020 | -0.047 | 0.420 | 0.002 | -0.030 | 0.533 | 0.001 | -0.050 | 0.287 | 0.003 |
| DLG4 | rs11650232 |  | -0.016 | 0.673 | 0.000 | 0.031 | 0.671 | 0.001 | 0.017 | 0.770 | 0.000 | 0.003 | 0.942 | 0.000 | 0.006 | 0.903 | 0.000 |
| DLG4 | rs314253 |  | 0.020 | 0.617 | 0.000 | -0.079 | 0.290 | 0.006 | -0.029 | 0.636 | 0.001 | -0.019 | 0.677 | 0.000 | -0.019 | 0.684 | 0.000 |
| DLG4 | rs314252 |  | -0.029 | 0.437 | 0.001 | 0.037 | 0.601 | 0.001 | -0.076 | 0.200 | 0.006 | 0.049 | 0.298 | 0.002 | 0.006 | 0.900 | 0.000 |
| DLG4 | rs17203281 |  | 0.047 | 0.210 | 0.002 | -0.094 | 0.184 | 0.009 | -0.084 | 0.151 | 0.007 | -0.027 | 0.516 | 0.001 | -0.047 | 0.282 | 0.002 |
| DLG4 | rs3826408 |  | 0.102 | 0.010 | 0.010 | -0.203 | 0.017 | 0.041 | -0.084 | 0.171 | 0.007 | -0.049 | 0.251 | 0.002 | -0.059 | 0.206 | 0.003 |
| DLG4 | rs1875673 |  | -0.074 | 0.065 | 0.005 | 0.227 | 0.009 | 0.052 | 0.059 | 0.357 | 0.003 | 0.043 | 0.335 | 0.002 | 0.056 | 0.228 | 0.003 |
| DLG4 | rs390200 |  | -0.079 | 0.048 | 0.006 | 0.184 | 0.032 | 0.034 | 0.014 | 0.825 | 0.000 | 0.050 | 0.255 | 0.003 | 0.051 | 0.272 | 0.003 |
| DLG4 | rs446994 |  | -0.081 | 0.047 | 0.007 | 0.171 | 0.048 | 0.029 | 0.005 | 0.942 | 0.000 | 0.039 | 0.374 | 0.002 | 0.048 | 0.306 | 0.002 |
| DLG4 | rs739669 |  | -0.078 | 0.056 | 0.006 | 0.214 | 0.015 | 0.046 | 0.050 | 0.439 | 0.003 | 0.045 | 0.308 | 0.002 | 0.058 | 0.218 | 0.003 |
| DLG4 | rs2017365 |  | -0.073 | 0.071 | 0.005 | 0.205 | 0.018 | 0.042 | 0.033 | 0.608 | 0.001 | 0.039 | 0.377 | 0.002 | 0.059 | 0.202 | 0.003 |
| DLG4 | rs2074222 |  | -0.077 | 0.058 | 0.006 | 0.217 | 0.014 | 0.047 | 0.037 | 0.564 | 0.001 | 0.043 | 0.339 | 0.002 | 0.066 | 0.154 | 0.004 |
| DLG4 | rs222837 |  | -0.022 | 0.567 | 0.000 | 0.145 | 0.065 | 0.021 | 0.006 | 0.918 | 0.000 | 0.056 | 0.216 | 0.003 | 0.027 | 0.550 | 0.001 |
| DLGAP2 | rs4876080 |  | -0.011 | 0.785 | 0.000 | -0.034 | 0.623 | 0.001 | -0.018 | 0.756 | 0.000 | 0.030 | 0.517 | 0.001 | 0.003 | 0.953 | 0.000 |
| DLGAP2 | rs2019513 |  | 0.015 | 0.707 | 0.000 | -0.076 | 0.274 | 0.006 | -0.005 | 0.922 | 0.000 | -0.013 | 0.740 | 0.000 | -0.063 | 0.120 | 0.004 |
| DLGAP2 | rs2956913 |  | -0.012 | 0.753 | 0.000 | 0.036 | 0.622 | 0.001 | -0.043 | 0.461 | 0.002 | 0.044 | 0.320 | 0.002 | 0.026 | 0.571 | 0.001 |
| DLGAP2 | rs10448097 |  | 0.044 | 0.247 | 0.002 | -0.061 | 0.401 | 0.004 | -0.092 | 0.105 | 0.008 | -0.004 | 0.921 | 0.000 | 0.020 | 0.643 | 0.000 |
| DLGAP2 | rs6992443 |  | 0.064 | 0.086 | 0.004 | -0.135 | 0.058 | 0.018 | -0.042 | 0.444 | 0.002 | -0.045 | 0.239 | 0.002 | -0.081 | 0.048 | 0.007 |
| DLGAP4 | rs6124857 |  | 0.045 | 0.257 | 0.002 | -0.067 | 0.378 | 0.004 | -0.007 | 0.905 | 0.000 | 0.069 | 0.124 | 0.005 | 0.037 | 0.441 | 0.001 |
| FKBP5 | rs3800374 |  | -0.010 | 0.786 | 0.000 | -0.074 | 0.322 | 0.005 | -0.008 | 0.900 | 0.000 | -0.053 | 0.250 | 0.003 | -0.003 | 0.942 | 0.000 |
| FKBP5 | rs10807151 |  | -0.008 | 0.826 | 0.000 | -0.072 | 0.328 | 0.005 | 0.006 | 0.918 | 0.000 | -0.042 | 0.339 | 0.002 | 0.012 | 0.787 | 0.000 |
| FKBP5 | rs3800373 |  | -0.016 | 0.676 | 0.000 | -0.068 | 0.365 | 0.005 | -0.002 | 0.974 | 0.000 | -0.072 | 0.110 | 0.005 | 0.052 | 0.254 | 0.003 |
| FKBP5 | rs7757037 |  | 0.018 | 0.632 | 0.000 | 0.079 | 0.272 | 0.006 | -0.052 | 0.379 | 0.003 | 0.058 | 0.196 | 0.003 | 0.039 | 0.384 | 0.002 |
| FKBP5 | rs9380524 |  | 0.042 | 0.280 | 0.002 | -0.059 | 0.394 | 0.003 | -0.014 | 0.813 | 0.000 | -0.022 | 0.605 | 0.000 | -0.051 | 0.278 | 0.003 |
| FKBP5 | rs7748266 |  | -0.012 | 0.754 | 0.000 | -0.081 | 0.281 | 0.007 | -0.016 | 0.793 | 0.000 | -0.030 | 0.520 | 0.001 | -0.024 | 0.606 | 0.001 |
| FKBP5 | rs1360780 |  | -0.004 | 0.914 | 0.000 | -0.096 | 0.205 | 0.009 | -0.011 | 0.851 | 0.000 | -0.072 | 0.107 | 0.005 | 0.024 | 0.587 | 0.001 |
| FKBP5 | rs1334894 |  | -0.002 | 0.953 | 0.000 | -0.096 | 0.257 | 0.009 | -0.061 | 0.363 | 0.004 | -0.024 | 0.603 | 0.001 | 0.010 | 0.827 | 0.000 |
| FKBP5 | rs9380525 |  | 0.009 | 0.810 | 0.000 | -0.071 | 0.355 | 0.005 | -0.006 | 0.926 | 0.000 | -0.060 | 0.178 | 0.004 | 0.008 | 0.860 | 0.000 |
| FKBP5 | rs9368881 |  | 0.004 | 0.922 | 0.000 | -0.060 | 0.428 | 0.004 | -0.002 | 0.978 | 0.000 | -0.059 | 0.181 | 0.003 | 0.011 | 0.806 | 0.000 |
| FKBP5 | rs4713916 |  | -0.025 | 0.506 | 0.001 | -0.057 | 0.461 | 0.003 | -0.003 | 0.967 | 0.000 | -0.023 | 0.612 | 0.001 | -0.012 | 0.799 | 0.000 |
| FKBP5 | rs2395635 |  | -0.021 | 0.577 | 0.000 | -0.074 | 0.341 | 0.005 | -0.009 | 0.884 | 0.000 | -0.026 | 0.577 | 0.001 | -0.012 | 0.792 | 0.000 |
| FKBP5 | rs2766534 |  | 0.016 | 0.682 | 0.000 | 0.073 | 0.288 | 0.005 | 0.015 | 0.778 | 0.000 | 0.011 | 0.799 | 0.000 | 0.027 | 0.556 | 0.001 |
| GNB1 | rs9660180 |  | 0.005 | 0.890 | 0.000 | 0.085 | 0.270 | 0.007 | -0.166 | 0.006 | 0.028 | 0.014 | 0.753 | 0.000 | -0.029 | 0.540 | 0.001 |
| GNB1 | rs6603803 |  | 0.013 | 0.740 | 0.000 | 0.084 | 0.269 | 0.007 | -0.172 | 0.004 | 0.030 | 0.013 | 0.772 | 0.000 | -0.031 | 0.509 | 0.001 |
| GRIA1 | rs1864205 |  | 0.040 | 0.297 | 0.002 | -0.146 | 0.059 | 0.021 | -0.039 | 0.485 | 0.002 | -0.068 | 0.132 | 0.005 | -0.052 | 0.270 | 0.003 |
| GRIA1 | rs7727515 |  | 0.058 | 0.136 | 0.003 | -0.135 | 0.077 | 0.018 | -0.018 | 0.765 | 0.000 | -0.014 | 0.738 | 0.000 | -0.061 | 0.189 | 0.004 |
| GRIA1 | rs9324750 |  | -0.017 | 0.663 | 0.000 | 0.054 | 0.476 | 0.003 | 0.019 | 0.751 | 0.000 | 0.006 | 0.904 | 0.000 | -0.033 | 0.480 | 0.001 |
| GRIA1 | rs12522802 |  | 0.019 | 0.615 | 0.000 | -0.001 | 0.987 | 0.000 | 0.103 | 0.069 | 0.011 | -0.005 | 0.900 | 0.000 | -0.090 | 0.045 | 0.008 |
| GRIA1 | rs12515520 |  | 0.007 | 0.853 | 0.000 | -0.005 | 0.949 | 0.000 | 0.101 | 0.075 | 0.010 | 0.011 | 0.806 | 0.000 | -0.058 | 0.201 | 0.003 |
| GRIA1 | rs17519810 |  | 0.084 | 0.022 | 0.007 | -0.035 | 0.564 | 0.001 | 0.034 | 0.530 | 0.001 | 0.026 | 0.517 | 0.001 | -0.001 | 0.988 | 0.000 |
| GRIA1 | rs1994862 |  | 0.003 | 0.933 | 0.000 | 0.018 | 0.819 | 0.000 | -0.045 | 0.446 | 0.002 | 0.006 | 0.891 | 0.000 | 0.022 | 0.632 | 0.000 |
| GRIA1 | rs1422884 |  | 0.034 | 0.381 | 0.001 | -0.025 | 0.731 | 0.001 | -0.013 | 0.816 | 0.000 | 0.009 | 0.840 | 0.000 | -0.101 | 0.032 | 0.010 |
| GRIA1 | rs10515697 |  | 0.005 | 0.911 | 0.000 | 0.019 | 0.813 | 0.000 | -0.045 | 0.444 | 0.002 | 0.005 | 0.909 | 0.000 | 0.023 | 0.623 | 0.001 |
| GRIA1 | rs2963954 |  | -0.019 | 0.630 | 0.000 | -0.025 | 0.732 | 0.001 | -0.031 | 0.587 | 0.001 | 0.030 | 0.512 | 0.001 | -0.025 | 0.599 | 0.001 |
| GRIA1 | rs707176 |  | -0.041 | 0.282 | 0.002 | 0.140 | 0.057 | 0.020 | 0.095 | 0.099 | 0.009 | 0.027 | 0.542 | 0.001 | 0.071 | 0.109 | 0.005 |
| GRIA1 | rs2910258 |  | -0.025 | 0.519 | 0.001 | 0.125 | 0.098 | 0.016 | 0.088 | 0.123 | 0.008 | 0.042 | 0.333 | 0.002 | 0.083 | 0.071 | 0.007 |
| GRIA1 | rs4385264 |  | 0.045 | 0.238 | 0.002 | -0.052 | 0.451 | 0.003 | 0.000 | 0.995 | 0.000 | 0.010 | 0.819 | 0.000 | 0.022 | 0.625 | 0.000 |
| GRIA1 | rs7714428 |  | -0.004 | 0.909 | 0.000 | -0.021 | 0.775 | 0.000 | -0.080 | 0.190 | 0.006 | -0.051 | 0.246 | 0.003 | -0.001 | 0.976 | 0.000 |
| GRIA1 | rs11741511 |  | 0.026 | 0.503 | 0.001 | -0.006 | 0.933 | 0.000 | 0.025 | 0.672 | 0.001 | 0.006 | 0.886 | 0.000 | 0.041 | 0.347 | 0.002 |
| GRIA1 | rs4077374 |  | 0.010 | 0.788 | 0.000 | 0.027 | 0.689 | 0.001 | 0.039 | 0.505 | 0.002 | 0.013 | 0.769 | 0.000 | 0.048 | 0.277 | 0.002 |
| GRIA1 | rs17594729 |  | 0.030 | 0.478 | 0.001 | -0.064 | 0.407 | 0.004 | -0.010 | 0.875 | 0.000 | 0.009 | 0.836 | 0.000 | -0.027 | 0.576 | 0.001 |
| GRIA1 | rs11953799 |  | -0.012 | 0.755 | 0.000 | 0.019 | 0.787 | 0.000 | 0.020 | 0.730 | 0.000 | 0.046 | 0.282 | 0.002 | -0.041 | 0.351 | 0.002 |
| GRIA1 | rs10035143 |  | -0.015 | 0.709 | 0.000 | 0.043 | 0.532 | 0.002 | 0.055 | 0.289 | 0.003 | 0.007 | 0.864 | 0.000 | -0.005 | 0.914 | 0.000 |
| GRIN2A | rs1014531 |  | 0.006 | 0.869 | 0.000 | -0.065 | 0.368 | 0.004 | -0.021 | 0.709 | 0.000 | -0.064 | 0.159 | 0.004 | -0.045 | 0.304 | 0.002 |
| GRIN2A | rs7190785 |  | -0.025 | 0.518 | 0.001 | 0.039 | 0.595 | 0.002 | 0.064 | 0.307 | 0.004 | 0.097 | 0.029 | 0.009 | 0.022 | 0.645 | 0.000 |
| GRIN2A | rs1544604 |  | 0.008 | 0.839 | 0.000 | -0.054 | 0.443 | 0.003 | -0.113 | 0.045 | 0.013 | -0.051 | 0.242 | 0.003 | -0.027 | 0.535 | 0.001 |
| GRIN2A | rs8050843 |  | 0.000 | 0.993 | 0.000 | -0.035 | 0.607 | 0.001 | -0.090 | 0.095 | 0.008 | -0.074 | 0.087 | 0.005 | -0.021 | 0.617 | 0.000 |
| GRIN2A | rs2215718 |  | 0.010 | 0.787 | 0.000 | -0.012 | 0.856 | 0.000 | -0.066 | 0.224 | 0.004 | -0.039 | 0.372 | 0.002 | -0.024 | 0.565 | 0.001 |
| GRIN2A | rs1548808 |  | -0.047 | 0.221 | 0.002 | 0.069 | 0.331 | 0.005 | -0.022 | 0.688 | 0.000 | 0.046 | 0.280 | 0.002 | 0.072 | 0.120 | 0.005 |
| GRIN2A | rs9931155 |  | 0.002 | 0.952 | 0.000 | -0.015 | 0.825 | 0.000 | -0.011 | 0.837 | 0.000 | -0.039 | 0.368 | 0.002 | -0.026 | 0.549 | 0.001 |
| GRIN2A | rs4782039 |  | 0.016 | 0.679 | 0.000 | -0.024 | 0.740 | 0.001 | 0.016 | 0.783 | 0.000 | 0.051 | 0.218 | 0.003 | -0.055 | 0.227 | 0.003 |
| GRIN2A | rs13331097 |  | 0.002 | 0.953 | 0.000 | 0.053 | 0.474 | 0.003 | 0.050 | 0.401 | 0.003 | 0.006 | 0.897 | 0.000 | 0.062 | 0.186 | 0.004 |
| GRIN2A | rs3859123 |  | -0.021 | 0.597 | 0.000 | -0.120 | 0.122 | 0.014 | -0.128 | 0.041 | 0.016 | -0.055 | 0.251 | 0.003 | -0.026 | 0.576 | 0.001 |
| GRIN2A | rs837694 |  | -0.026 | 0.497 | 0.001 | 0.078 | 0.266 | 0.006 | -0.016 | 0.775 | 0.000 | 0.049 | 0.248 | 0.002 | -0.032 | 0.473 | 0.001 |
| GRIN2A | rs11074568 |  | -0.014 | 0.724 | 0.000 | -0.028 | 0.691 | 0.001 | 0.036 | 0.508 | 0.001 | -0.044 | 0.310 | 0.002 | -0.012 | 0.791 | 0.000 |
| GRIN2A | rs10438517 |  | 0.004 | 0.918 | 0.000 | 0.066 | 0.387 | 0.004 | 0.026 | 0.649 | 0.001 | -0.004 | 0.925 | 0.000 | -0.061 | 0.212 | 0.004 |
| GRIN2A | rs7499321 |  | 0.017 | 0.660 | 0.000 | 0.019 | 0.791 | 0.000 | -0.075 | 0.198 | 0.006 | -0.041 | 0.347 | 0.002 | -0.048 | 0.304 | 0.002 |
| GRIN2A | rs1420666 |  | -0.007 | 0.851 | 0.000 | 0.043 | 0.533 | 0.002 | 0.042 | 0.444 | 0.002 | 0.046 | 0.292 | 0.002 | 0.038 | 0.384 | 0.001 |
| GRIN2B | rs1806213 |  | -0.002 | 0.961 | 0.000 | 0.016 | 0.839 | 0.000 | -0.048 | 0.451 | 0.002 | 0.022 | 0.636 | 0.000 | -0.010 | 0.833 | 0.000 |
| GRIN2B | rs1805513 |  | 0.020 | 0.599 | 0.000 | -0.091 | 0.202 | 0.008 | 0.055 | 0.356 | 0.003 | -0.025 | 0.575 | 0.001 | -0.081 | 0.076 | 0.007 |
| GRIN2B | rs12321321 |  | 0.012 | 0.743 | 0.000 | 0.007 | 0.920 | 0.000 | -0.061 | 0.295 | 0.004 | 0.009 | 0.840 | 0.000 | -0.057 | 0.206 | 0.003 |
| GRIN2B | rs2300250 |  | -0.038 | 0.338 | 0.001 | 0.023 | 0.754 | 0.001 | 0.103 | 0.092 | 0.011 | 0.060 | 0.177 | 0.004 | 0.006 | 0.902 | 0.000 |
| GRIN2B | rs4763358 |  | -0.057 | 0.158 | 0.003 | 0.113 | 0.151 | 0.013 | 0.097 | 0.104 | 0.009 | 0.100 | 0.029 | 0.010 | 0.049 | 0.288 | 0.002 |
| GRIN2B | rs1075010 |  | 0.029 | 0.458 | 0.001 | -0.054 | 0.473 | 0.003 | -0.090 | 0.137 | 0.008 | -0.026 | 0.560 | 0.001 | -0.066 | 0.144 | 0.004 |
| GRIN2B | rs7301500 |  | 0.021 | 0.599 | 0.000 | -0.068 | 0.371 | 0.005 | -0.034 | 0.580 | 0.001 | -0.081 | 0.074 | 0.007 | -0.019 | 0.704 | 0.000 |
| GRIN2B | rs17833967 |  | -0.036 | 0.348 | 0.001 | 0.155 | 0.065 | 0.024 | 0.096 | 0.107 | 0.009 | 0.057 | 0.209 | 0.003 | 0.002 | 0.972 | 0.000 |
| GRIN2B | rs10772719 |  | -0.027 | 0.472 | 0.001 | 0.095 | 0.171 | 0.009 | 0.108 | 0.063 | 0.012 | 0.078 | 0.061 | 0.006 | 0.056 | 0.194 | 0.003 |
| GRIN2B | rs12824372 |  | 0.020 | 0.603 | 0.000 | 0.014 | 0.847 | 0.000 | 0.032 | 0.570 | 0.001 | 0.026 | 0.568 | 0.001 | 0.015 | 0.741 | 0.000 |
| GRIN2B | rs17339365 |  | -0.010 | 0.804 | 0.000 | 0.012 | 0.870 | 0.000 | -0.035 | 0.541 | 0.001 | -0.008 | 0.848 | 0.000 | 0.082 | 0.076 | 0.007 |
| GRM3 | rs802432 |  | 0.054 | 0.141 | 0.003 | -0.095 | 0.148 | 0.009 | 0.027 | 0.632 | 0.001 | -0.003 | 0.945 | 0.000 | -0.057 | 0.190 | 0.003 |
| GRM3 | rs802434 |  | 0.003 | 0.928 | 0.000 | -0.080 | 0.237 | 0.006 | -0.077 | 0.142 | 0.006 | -0.054 | 0.212 | 0.003 | -0.076 | 0.083 | 0.006 |
| GRM3 | rs2228595 |  | -0.059 | 0.106 | 0.003 | 0.109 | 0.130 | 0.012 | 0.102 | 0.072 | 0.010 | 0.047 | 0.273 | 0.002 | 0.095 | 0.053 | 0.009 |
| GRM3 | rs1468412 |  | -0.040 | 0.293 | 0.002 | 0.104 | 0.142 | 0.011 | 0.109 | 0.051 | 0.012 | 0.069 | 0.105 | 0.005 | 0.018 | 0.707 | 0.000 |
| GRM3 | rs2282966 |  | -0.018 | 0.651 | 0.000 | 0.041 | 0.585 | 0.002 | 0.050 | 0.407 | 0.003 | 0.009 | 0.828 | 0.000 | 0.005 | 0.913 | 0.000 |
| IQSEC3 | rs7294904 |  | -0.027 | 0.496 | 0.001 | -0.011 | 0.883 | 0.000 | -0.002 | 0.966 | 0.000 | 0.053 | 0.215 | 0.003 | 0.004 | 0.929 | 0.000 |
| IQSEC3 | rs2368785 |  | -0.014 | 0.727 | 0.000 | -0.040 | 0.578 | 0.002 | 0.016 | 0.786 | 0.000 | 0.042 | 0.353 | 0.002 | -0.007 | 0.876 | 0.000 |
| LRRC7 | rs1340769 |  | -0.038 | 0.329 | 0.001 | -0.008 | 0.918 | 0.000 | 0.012 | 0.830 | 0.000 | -0.025 | 0.593 | 0.001 | 0.044 | 0.350 | 0.002 |
| LRRC7 | rs6697066 |  | -0.015 | 0.693 | 0.000 | 0.040 | 0.573 | 0.002 | 0.009 | 0.876 | 0.000 | 0.015 | 0.738 | 0.000 | -0.002 | 0.969 | 0.000 |
| LRRC7 | rs10789303 |  | 0.042 | 0.265 | 0.002 | -0.039 | 0.575 | 0.002 | 0.040 | 0.495 | 0.002 | 0.001 | 0.976 | 0.000 | 0.001 | 0.989 | 0.000 |
| LRRC7 | rs1913268 |  | -0.002 | 0.967 | 0.000 | 0.006 | 0.929 | 0.000 | 0.016 | 0.776 | 0.000 | 0.043 | 0.327 | 0.002 | -0.002 | 0.959 | 0.000 |
| LRRC7 | rs12033206 |  | -0.014 | 0.722 | 0.000 | -0.141 | 0.063 | 0.020 | -0.030 | 0.599 | 0.001 | -0.045 | 0.300 | 0.002 | -0.006 | 0.890 | 0.000 |
| LRRC7 | rs12136628 |  | -0.018 | 0.648 | 0.000 | -0.039 | 0.605 | 0.002 | -0.013 | 0.818 | 0.000 | -0.018 | 0.702 | 0.000 | 0.036 | 0.460 | 0.001 |
| NR3C1 | rs17209237 |  | -0.029 | 0.458 | 0.001 | 0.080 | 0.283 | 0.006 | 0.022 | 0.687 | 0.000 | 0.008 | 0.861 | 0.000 | 0.101 | 0.035 | 0.010 |
| NR3C1 | rs6196 |  | -0.005 | 0.898 | 0.000 | -0.130 | 0.079 | 0.017 | -0.058 | 0.360 | 0.003 | 0.020 | 0.635 | 0.000 | -0.038 | 0.378 | 0.001 |
| NR3C1 | rs852977 |  | 0.074 | 0.058 | 0.005 | -0.199 | 0.013 | 0.040 | -0.148 | 0.014 | 0.022 | -0.049 | 0.277 | 0.002 | -0.086 | 0.064 | 0.007 |
| NR3C1 | rs860457 |  | 0.074 | 0.058 | 0.005 | -0.199 | 0.013 | 0.040 | -0.148 | 0.014 | 0.022 | -0.049 | 0.277 | 0.002 | -0.086 | 0.064 | 0.007 |
| NR3C1 | rs10482689 |  | 0.086 | 0.028 | 0.007 | -0.121 | 0.129 | 0.015 | -0.115 | 0.053 | 0.013 | -0.068 | 0.145 | 0.005 | -0.055 | 0.265 | 0.003 |
| NR3C1 | rs41423247 |  | -0.032 | 0.410 | 0.001 | -0.023 | 0.754 | 0.001 | -0.021 | 0.709 | 0.000 | 0.018 | 0.678 | 0.000 | 0.060 | 0.206 | 0.004 |
| NR3C1 | rs10482605 |  | 0.072 | 0.061 | 0.005 | -0.084 | 0.280 | 0.007 | -0.081 | 0.165 | 0.007 | -0.061 | 0.174 | 0.004 | -0.048 | 0.314 | 0.002 |
| NR3C1 | rs10052957 |  | 0.067 | 0.081 | 0.004 | -0.194 | 0.015 | 0.038 | -0.127 | 0.036 | 0.016 | -0.046 | 0.294 | 0.002 | -0.082 | 0.074 | 0.007 |
| NR3C2 | rs17620330 |  | 0.011 | 0.767 | 0.000 | 0.020 | 0.783 | 0.000 | 0.060 | 0.290 | 0.004 | -0.042 | 0.343 | 0.002 | 0.072 | 0.098 | 0.005 |
| NR3C2 | rs10519959 |  | -0.044 | 0.249 | 0.002 | -0.008 | 0.903 | 0.000 | -0.021 | 0.710 | 0.000 | -0.023 | 0.610 | 0.001 | 0.025 | 0.587 | 0.001 |
| NR3C2 | rs6831034 |  | -0.018 | 0.639 | 0.000 | -0.037 | 0.618 | 0.001 | -0.018 | 0.765 | 0.000 | -0.009 | 0.847 | 0.000 | 0.012 | 0.803 | 0.000 |
| NR3C2 | rs5522 |  | 0.019 | 0.630 | 0.000 | -0.007 | 0.919 | 0.000 | -0.008 | 0.899 | 0.000 | 0.048 | 0.273 | 0.002 | 0.069 | 0.091 | 0.005 |
| NR3C2 | rs2070951 |  | 0.062 | 0.106 | 0.004 | -0.002 | 0.982 | 0.000 | -0.011 | 0.847 | 0.000 | -0.063 | 0.162 | 0.004 | -0.131 | 0.004 | 0.017 |
| NRG1 | rs35753505 |  | -0.005 | 0.909 | 0.000 | -0.027 | 0.719 | 0.001 | 0.009 | 0.879 | 0.000 | 0.069 | 0.120 | 0.005 | 0.054 | 0.261 | 0.003 |
| NRG1 | rs4733264 |  | 0.023 | 0.563 | 0.001 | -0.123 | 0.115 | 0.015 | -0.010 | 0.863 | 0.000 | 0.060 | 0.181 | 0.004 | 0.021 | 0.658 | 0.000 |
| NRG1 | rs6987996 |  | 0.016 | 0.681 | 0.000 | -0.042 | 0.578 | 0.002 | -0.016 | 0.778 | 0.000 | -0.062 | 0.155 | 0.004 | -0.081 | 0.083 | 0.007 |
| NRG1 | rs4552856 |  | -0.010 | 0.799 | 0.000 | 0.013 | 0.857 | 0.000 | -0.036 | 0.576 | 0.001 | -0.052 | 0.257 | 0.003 | 0.009 | 0.836 | 0.000 |
| NRG1 | rs13268778 |  | -0.020 | 0.608 | 0.000 | 0.022 | 0.756 | 0.000 | -0.041 | 0.504 | 0.002 | -0.037 | 0.427 | 0.001 | 0.034 | 0.456 | 0.001 |
| NRG1 | rs12679454 |  | 0.062 | 0.100 | 0.004 | 0.025 | 0.691 | 0.001 | -0.051 | 0.326 | 0.003 | -0.015 | 0.706 | 0.000 | -0.048 | 0.246 | 0.002 |
| NRG1 | rs16878368 |  | -0.050 | 0.200 | 0.003 | 0.058 | 0.431 | 0.003 | 0.020 | 0.747 | 0.000 | 0.091 | 0.034 | 0.008 | 0.061 | 0.191 | 0.004 |
| NRG1 | rs2683771 |  | 0.077 | 0.052 | 0.006 | -0.148 | 0.059 | 0.022 | -0.068 | 0.272 | 0.005 | 0.004 | 0.936 | 0.000 | -0.050 | 0.300 | 0.003 |
| NRG1 | rs776382 |  | 0.088 | 0.024 | 0.008 | -0.190 | 0.017 | 0.036 | -0.051 | 0.390 | 0.003 | -0.042 | 0.356 | 0.002 | -0.070 | 0.143 | 0.005 |
| NRG1 | rs16878644 |  | 0.062 | 0.106 | 0.004 | 0.053 | 0.400 | 0.003 | 0.004 | 0.934 | 0.000 | 0.000 | 0.995 | 0.000 | -0.072 | 0.107 | 0.005 |
| NRG1 | rs2068226 |  | 0.031 | 0.429 | 0.001 | 0.026 | 0.727 | 0.001 | -0.010 | 0.872 | 0.000 | 0.047 | 0.302 | 0.002 | 0.010 | 0.826 | 0.000 |
| NRG1 | rs6468091 |  | 0.012 | 0.766 | 0.000 | 0.022 | 0.763 | 0.000 | -0.039 | 0.503 | 0.002 | -0.003 | 0.949 | 0.000 | -0.004 | 0.932 | 0.000 |
| NRG1 | rs17624670 |  | -0.013 | 0.737 | 0.000 | -0.024 | 0.751 | 0.001 | -0.095 | 0.116 | 0.009 | 0.023 | 0.605 | 0.001 | 0.006 | 0.891 | 0.000 |
| NRG1 | rs17624997 |  | 0.004 | 0.916 | 0.000 | -0.113 | 0.134 | 0.013 | -0.108 | 0.095 | 0.012 | -0.029 | 0.516 | 0.001 | -0.052 | 0.252 | 0.003 |
| NRG1 | rs6468099 |  | -0.051 | 0.229 | 0.003 | 0.055 | 0.500 | 0.003 | -0.094 | 0.138 | 0.009 | 0.032 | 0.504 | 0.001 | 0.072 | 0.143 | 0.005 |
| NRG1 | rs16879327 |  | 0.027 | 0.462 | 0.001 | 0.115 | 0.103 | 0.013 | 0.075 | 0.156 | 0.006 | 0.007 | 0.865 | 0.000 | 0.082 | 0.073 | 0.007 |
| NRG1 | rs12334435 |  | 0.030 | 0.439 | 0.001 | -0.016 | 0.825 | 0.000 | 0.004 | 0.943 | 0.000 | 0.067 | 0.127 | 0.004 | 0.004 | 0.924 | 0.000 |
| NRG1 | rs7826312 |  | -0.011 | 0.786 | 0.000 | -0.022 | 0.765 | 0.000 | 0.013 | 0.826 | 0.000 | 0.045 | 0.307 | 0.002 | -0.017 | 0.718 | 0.000 |
| NRG1 | rs2466103 |  | -0.014 | 0.703 | 0.000 | -0.055 | 0.425 | 0.003 | 0.023 | 0.667 | 0.001 | 0.005 | 0.917 | 0.000 | -0.018 | 0.682 | 0.000 |
| NRG1 | rs2439292 |  | -0.003 | 0.939 | 0.000 | -0.010 | 0.888 | 0.000 | -0.071 | 0.217 | 0.005 | 0.035 | 0.414 | 0.001 | 0.065 | 0.140 | 0.004 |
| NRG1 | rs2439318 |  | 0.027 | 0.507 | 0.001 | -0.128 | 0.125 | 0.016 | 0.012 | 0.846 | 0.000 | 0.094 | 0.040 | 0.009 | -0.038 | 0.414 | 0.001 |
| NRG1 | rs12546380 |  | 0.005 | 0.906 | 0.000 | -0.013 | 0.861 | 0.000 | 0.029 | 0.624 | 0.001 | -0.019 | 0.667 | 0.000 | 0.010 | 0.823 | 0.000 |
| NRG1 | rs6468122 |  | 0.004 | 0.918 | 0.000 | -0.080 | 0.320 | 0.006 | 0.019 | 0.761 | 0.000 | 0.058 | 0.199 | 0.003 | -0.009 | 0.859 | 0.000 |
| NRG1 | rs10503929 |  | -0.005 | 0.905 | 0.000 | -0.042 | 0.546 | 0.002 | 0.063 | 0.271 | 0.004 | -0.055 | 0.210 | 0.003 | 0.000 | 0.993 | 0.000 |
| NRG1 | rs3735782 |  | -0.036 | 0.357 | 0.001 | -0.098 | 0.196 | 0.010 | 0.093 | 0.117 | 0.009 | -0.003 | 0.949 | 0.000 | -0.002 | 0.959 | 0.000 |
| SLC6A4 | rs1042173 |  | 0.026 | 0.504 | 0.001 | 0.019 | 0.784 | 0.000 | -0.017 | 0.791 | 0.000 | 0.047 | 0.270 | 0.002 | -0.021 | 0.653 | 0.000 |
| SLC6A4 | rs3794808 |  | 0.010 | 0.805 | 0.000 | 0.027 | 0.704 | 0.001 | -0.034 | 0.584 | 0.001 | 0.040 | 0.359 | 0.002 | -0.032 | 0.492 | 0.001 |
| SLC6A4 | rs140700 |  | -0.009 | 0.819 | 0.000 | -0.054 | 0.435 | 0.003 | 0.019 | 0.720 | 0.000 | -0.034 | 0.449 | 0.001 | 0.014 | 0.739 | 0.000 |
| SLC6A4 | rs2020936 |  | -0.021 | 0.600 | 0.000 | -0.077 | 0.318 | 0.006 | -0.020 | 0.733 | 0.000 | 0.002 | 0.962 | 0.000 | 0.013 | 0.774 | 0.000 |
| SLC6A4 | rs4251417 |  | -0.013 | 0.717 | 0.000 | 0.071 | 0.315 | 0.005 | 0.071 | 0.206 | 0.005 | 0.019 | 0.672 | 0.000 | -0.033 | 0.475 | 0.001 |
| SLC6A4 | rs2020934 |  | -0.024 | 0.535 | 0.001 | 0.050 | 0.484 | 0.003 | -0.068 | 0.259 | 0.005 | 0.006 | 0.889 | 0.000 | 0.001 | 0.986 | 0.000 |
| SLC6A4 | rs2020933 |  | 0.008 | 0.822 | 0.000 | 0.049 | 0.480 | 0.002 | -0.032 | 0.600 | 0.001 | 0.045 | 0.349 | 0.002 | 0.033 | 0.464 | 0.001 |
| SYNGAP1 | rs211456 |  | -0.055 | 0.153 | 0.003 | 0.175 | 0.020 | 0.031 | 0.134 | 0.022 | 0.018 | 0.039 | 0.371 | 0.002 | 0.082 | 0.079 | 0.007 |
| SYNGAP1 | rs413722 |  | -0.032 | 0.421 | 0.001 | 0.014 | 0.851 | 0.000 | -0.010 | 0.863 | 0.000 | 0.039 | 0.374 | 0.002 | -0.025 | 0.605 | 0.001 |
| SYNGAP1 | rs9394145 |  | -0.003 | 0.941 | 0.000 | -0.042 | 0.595 | 0.002 | -0.116 | 0.071 | 0.013 | -0.070 | 0.151 | 0.005 | -0.013 | 0.794 | 0.000 |
| SYNGAP1 | rs411136 |  | -0.049 | 0.201 | 0.002 | 0.158 | 0.034 | 0.025 | 0.115 | 0.045 | 0.013 | 0.036 | 0.409 | 0.001 | 0.092 | 0.051 | 0.008 |
| SYNGAP1 | rs2247385 |  | -0.048 | 0.212 | 0.002 | 0.159 | 0.033 | 0.025 | 0.112 | 0.052 | 0.013 | 0.037 | 0.404 | 0.001 | 0.089 | 0.059 | 0.008 |
| TNIK | rs12486818 |  | -0.001 | 0.972 | 0.000 | 0.020 | 0.787 | 0.000 | 0.032 | 0.576 | 0.001 | 0.050 | 0.264 | 0.003 | -0.003 | 0.948 | 0.000 |
| TNIK | rs12488990 |  | -0.016 | 0.674 | 0.000 | -0.024 | 0.743 | 0.001 | 0.043 | 0.452 | 0.002 | -0.014 | 0.752 | 0.000 | 0.012 | 0.784 | 0.000 |
| TNIK | rs6444965 |  | -0.005 | 0.905 | 0.000 | -0.025 | 0.727 | 0.001 | 0.090 | 0.125 | 0.008 | -0.005 | 0.912 | 0.000 | 0.027 | 0.554 | 0.001 |
| TNIK | rs2292005 |  | 0.011 | 0.772 | 0.000 | -0.004 | 0.956 | 0.000 | 0.076 | 0.178 | 0.006 | -0.055 | 0.188 | 0.003 | -0.001 | 0.973 | 0.000 |
| TNIK | rs12637875 |  | 0.014 | 0.717 | 0.000 | -0.012 | 0.854 | 0.000 | 0.069 | 0.224 | 0.005 | -0.041 | 0.327 | 0.002 | -0.002 | 0.956 | 0.000 |
| TNIK | rs13098316 |  | 0.017 | 0.666 | 0.000 | -0.020 | 0.780 | 0.000 | -0.064 | 0.291 | 0.004 | 0.056 | 0.195 | 0.003 | 0.028 | 0.537 | 0.001 |
| TNIK | rs7618166 |  | 0.006 | 0.883 | 0.000 | 0.024 | 0.743 | 0.001 | -0.064 | 0.317 | 0.004 | 0.041 | 0.344 | 0.002 | 0.055 | 0.234 | 0.003 |
| TNIK | rs11716029 |  | -0.034 | 0.391 | 0.001 | 0.079 | 0.287 | 0.006 | -0.077 | 0.236 | 0.006 | 0.057 | 0.193 | 0.003 | 0.064 | 0.164 | 0.004 |
| TNIK | rs905129 |  | 0.026 | 0.515 | 0.001 | -0.012 | 0.863 | 0.000 | -0.007 | 0.907 | 0.000 | -0.031 | 0.486 | 0.001 | -0.054 | 0.238 | 0.003 |
| TNIK | rs9844004 |  | 0.052 | 0.190 | 0.003 | -0.034 | 0.650 | 0.001 | 0.021 | 0.704 | 0.000 | 0.017 | 0.708 | 0.000 | -0.060 | 0.212 | 0.004 |
| TNIK | rs16856044 |  | -0.025 | 0.545 | 0.001 | 0.037 | 0.632 | 0.001 | 0.062 | 0.247 | 0.004 | 0.007 | 0.876 | 0.000 | 0.022 | 0.649 | 0.000 |
| TNIK | rs7615457 |  | -0.025 | 0.527 | 0.001 | 0.052 | 0.495 | 0.003 | 0.041 | 0.502 | 0.002 | 0.075 | 0.103 | 0.006 | 0.042 | 0.366 | 0.002 |
| TNIK | rs2088885 |  | -0.057 | 0.150 | 0.003 | 0.078 | 0.313 | 0.006 | 0.054 | 0.372 | 0.003 | 0.081 | 0.077 | 0.007 | 0.060 | 0.202 | 0.004 |
| TNIK | rs7627954 |  | -0.058 | 0.145 | 0.003 | 0.081 | 0.298 | 0.007 | 0.051 | 0.398 | 0.003 | 0.086 | 0.061 | 0.007 | 0.066 | 0.164 | 0.004 |
| TNIK | rs4894643 |  | 0.064 | 0.096 | 0.004 | -0.072 | 0.314 | 0.005 | -0.052 | 0.358 | 0.003 | -0.060 | 0.159 | 0.004 | -0.081 | 0.074 | 0.007 |
| TNIK | rs4894814 |  | 0.053 | 0.161 | 0.003 | -0.038 | 0.565 | 0.001 | 0.045 | 0.385 | 0.002 | 0.016 | 0.673 | 0.000 | -0.037 | 0.372 | 0.001 |
| TNIK | rs16856172 |  | -0.041 | 0.270 | 0.002 | 0.096 | 0.159 | 0.009 | 0.065 | 0.225 | 0.004 | 0.011 | 0.801 | 0.000 | 0.052 | 0.282 | 0.003 |
| TNIK | rs902956 |  | -0.042 | 0.268 | 0.002 | -0.023 | 0.745 | 0.001 | 0.125 | 0.028 | 0.016 | -0.011 | 0.808 | 0.000 | -0.028 | 0.529 | 0.001 |
