# Supplementary material for: Psychometric precision in phenotype definition is a useful step in molecular genetic investigation of psychiatric disorders
Source: Transl Psychiatry. 2015 Jun 30;5(6):e593–. doi: 10.1038/tp.2015.86 (PMC4490295; doi:10.1038/tp.2015.86)
Supplement: Supplementary Figure 1 [file tp201586x4.doc]

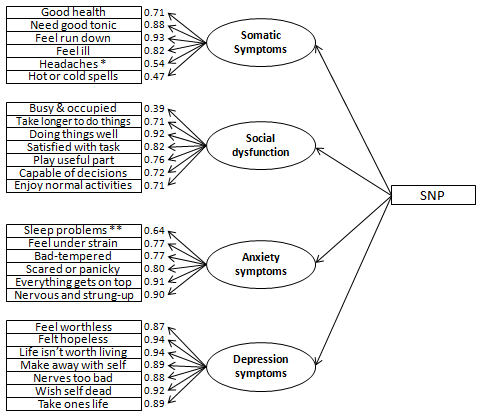


Figure S1. Psychometric model of GHQ-28 items with four first-order factors and a SNP predictor. Oval shapes are latent variables representing each affective disorder domains. Rectangular shapes are observed variables including both the SNP predictor and GHQ-28 items that are the basis of the latent variables. Arrows leading from a SNP variable to latent variables represent the regression path from the SNP predictor to global as well as specific phenotype dimensions. The arrows between the latent variable to the observed variables indicate the strength of the relationship between the two, represented by standardized factor loadings. The standardized factor loadings are based on a phenotype-only model (excluding the SNP predictor variable from the model). The fit indices of the CFA model without the SBP predictor were: 2116.267 (293 degree of freedom) for Chi-squared, 0.068 for RMSEA, 0.957 for CFI, and 0.952 for TLI.

* The indicator “headaches” was based on the sum of two highly correlated items. This is to avoid convergence problems caused by the high colinearity between the two items.

**Similarly, “sleep problems” was also based on two substantially correlated items. Both items were still treated as ordinal measures in model estimation.
