# Supplementary material for: Psychometric precision in phenotype definition is a useful step in molecular genetic investigation of psychiatric disorders
Source: Transl Psychiatry. 2015 Jun 30;5(6):e593–. doi: 10.1038/tp.2015.86 (PMC4490295; doi:10.1038/tp.2015.86)
Supplement: Supplementary Figure 2 [file tp201586x5.doc]

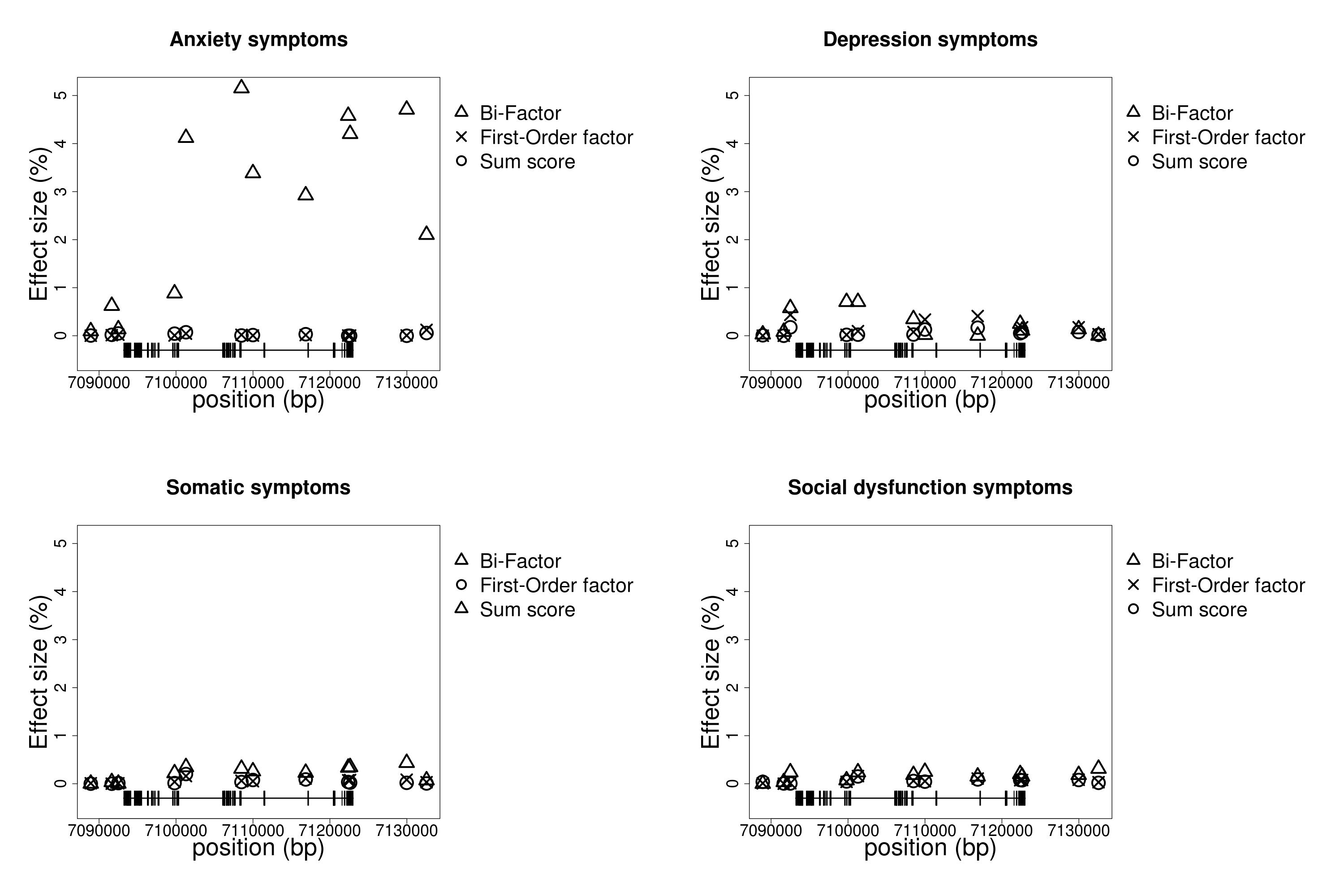


Figure S2. Association results for SNPs of the DLG4 gene (12 SNPs) for dimensional phenotypes from bi-factor approach, first-order factor model approach and sum score approach. The y-axis represents effect size in terms of the percentage of variance explained in the phenotype. The x-axis indicates the chromosome positions (bp). The bars at the bottom of the x-axis represent exon positions.
